# The cervicovaginal mucus barrier to HIV-1 is diminished in bacterial vaginosis

**DOI:** 10.1371/journal.ppat.1008236

**Published:** 2020-01-23

**Authors:** Thuy Hoang, Emily Toler, Kevin DeLong, Nomfuneko A. Mafunda, Seth M. Bloom, Hannah C. Zierden, Thomas R. Moench, Jenell S. Coleman, Justin Hanes, Douglas S. Kwon, Samuel K. Lai, Richard A. Cone, Laura M. Ensign

**Affiliations:** 1 The Center for Nanomedicine, The Wilmer Eye Institute, Johns Hopkins University School of Medicine, Baltimore, Maryland, United States of America; 2 Department of Pharmacology and Molecular Sciences, Johns Hopkins University School of Medicine, Baltimore, Maryland, United States of America; 3 Department of Ophthalmology, The Wilmer Eye Institute, Johns Hopkins University School of Medicine, Baltimore, Maryland, United States of America; 4 Ragon Institute of MGH, MIT, and Harvard, Massachusetts General Hospital, Cambridge, Massachusetts, United States of America; 5 Division of Infectious Diseases, Massachusetts General Hospital, Boston, Massachusetts, United States of America; 6 Harvard Medical School, Boston, Massachusetts, United States of America; 7 Department of Chemical and Biomolecular Engineering, Johns Hopkins University, Baltimore, Maryland, United States of America; 8 Department of Biophysics, Johns Hopkins University, Baltimore, Maryland, United States of America; 9 Department of Gynecology and Obstetrics, Johns Hopkins University School of Medicine, Baltimore, Maryland, United States of America; 10 The Sidney Kimmel Comprehensive Cancer Center at Johns Hopkins University, Baltimore, Maryland, United States of America; 11 Division of Pharmacoengineering and Molecular Pharmaceutics, Eshelman School of Pharmacy, UNC/NCSU Joint Department of Biomedical Engineering, Department of Microbiology & Immunology, University of North Carolina-Chapel Hill, Chapel Hill, North Carolina, United States of America; Emory University, UNITED STATES

## Abstract

Bacterial vaginosis (BV), a condition in which the vaginal microbiota consists of community of obligate and facultative anaerobes rather than dominated by a single species of *Lactobacillus*, affects ~30% of women in the US. Women with BV are at 60% increased risk for HIV acquisition and are 3-times more likely to transmit HIV to an uninfected partner. As cervicovaginal mucus (CVM) is the first line of defense against mucosal pathogens and the home of the resident vaginal microbiota, we hypothesized the barrier function of CVM to HIV may be diminished in BV. Here, we characterized CVM properties including pH, lactic acid content, and Nugent score to correlate with the microbiota community composition, which was confirmed by 16S rDNA sequencing on a subset of samples. We then quantified the mobility of fluorescently-labeled HIV virions and nanoparticles to characterize the structural and adhesive barrier properties of CVM. Our analyses included women with Nugent scores categorized as intermediate (4–6) and BV (7–10), women that were either symptomatic or asymptomatic, and a small group of women before and after antibiotic treatment for symptomatic BV. Overall, we found that HIV virions had significantly increased mobility in CVM from women with BV compared to CVM from women with *Lactobacillus crispatus*-dominant microbiota, regardless of whether symptoms were present. We confirmed using nanoparticles and scanning electron microscopy that the impaired barrier function was due to reduced adhesive barrier properties without an obvious degradation of the physical CVM pore structure. We further confirmed a similar increase in HIV mobility in CVM from women with *Lactobacillus iners*-dominant microbiota, the species most associated with transitions to BV and that persists after antibiotic treatment for BV. Our findings advance the understanding of the protective role of mucus and highlight the interplay between vaginal microbiota and the innate barrier function mucus.

## Author summary

Bacterial vaginosis (BV), a condition characterized by the depletion of lactobacillus bacteria in the vagina, is the most common vaginal condition in reproductive age women. BV has been associated with many adverse reproductive and sexual health outcomes, including increased risk of HIV infection. Cervicovaginal mucus is the home to vaginal bacteria and acts as a first line of defense to protect the underlying tissues and cells from infection. Here, we studied the barrier properties of mucus from women with BV compared to women with vaginal bacteria dominated by lactobacilli. We found that mucus from women with BV and women with *Lactobacillus iners* were permissive to HIV-1, which may allow the virus to more easily reach target cells. These findings are in agreement with the observed increased risk for HIV acquisition seen in women with BV and *L*. *iners* bacteria. Furthermore, we found that the barrier against HIV is diminished in women with BV regardless of whether they have symptoms. Our findings highlight the important, yet unexplored interactions between the mucus barrier and the vaginal microbiota and the implications for human health.

## Introduction

Bacterial vaginosis (BV) is a highly prevalent vaginal condition that affects approximately 30% of women in the US and as many as 44% of women in sub-Saharan Africa [1–3]. BV is associated with a multitude of obstetric and sexual health complications, including preterm birth, adverse neonatal outcomes, miscarriage, chorioamnionitis, endometritis, pelvic inflammatory disease (PID), urinary tract infection (UTI) and increased risk for sexually transmitted infections (STIs) acquisition/transmission, including human immunodeficiency virus (HIV) [4–7]. The vaginal microbiota in BV is polymicrobial, generally defined as depleted in *Lactobacillus* spp. with an increase in obligate anaerobes and/or facultative anaerobes [8–11]. The mechanisms by which BV, or similarly, the lack of *Lactobacillus* spp., negatively impacts such a wide range of sexual and reproductive outcomes are poorly understood.

It has been reported that women with BV are at 60% increased risk for HIV-1 acquisition [4]. In a recent prospective cohort of South African women, it was found that women with high bacterial diversity and low *Lactobacillus* abundance had a 4-fold increase in incident HIV infection compared to women with *Lactobacillus crispatus*-dominant vaginal microbiota [12]. Furthermore, in a cohort analysis of HIV-1 seropositive women, BV was associated with a 3-fold increased risk of female-to-male transmission [13]. Many hypotheses to explain the association between BV and HIV acquisition and transmission risk focus on the impact that vaginal bacteria have on tissue integrity, inflammation, elevation of pro-inflammatory cytokines/chemokines, and recruitment and activation of HIV target cells [12, 14, 15]. However, the first line of defense for the mucosa, and the home of the resident microbiota, is the mucus coating the cervicovaginal epithelium. Cervicovaginal mucus (CVM) both lubricates and protects the underlying epithelium, serving as the first line of defense against viral acquisition and transmission. It is known that the resident microbiota can modify the properties of mucus in the airways and gastrointestinal tract, and thus is likely of importance in the female reproductive tract [16–18]. Here, we aim to study the impact of polymicrobial vaginal microbiota on the structural and barrier properties of CVM to HIV.

We previously demonstrated that fluorescently-labeled HIV virions were trapped in CVM with low Nugent score and acidic pH (indicative of *Lactobacillus*-dominance) and that trapping was due to mucoadhesion rather than steric obstruction [19, 20]. We further showed that HSV-2 was adhesively trapped in *Lactobacillus*-dominant CVM [21], likely due to crosslinking with the mucins [22]. Others have reported similar findings that CVM and cervical mucus impedes the diffusion of HIV [23, 24]. Indeed, trapping by mucus has been shown to be an important mechanism for protecting the underlying epithelium from infectious pathogens. It was demonstrated in a murine model that increased trapping of HSV-2 in mucus led to increased protection against infection *in vivo* [22]. Similarly, Carias et al. showed that when *ex vivo* human cervical explants were inoculated with fluorescently-labeled HIV virions, many virions were bound to mucus and relatively few penetrated into the tissue [25]. They noted that either efferent mucus flow and/or hindrance by mucus could explain the reduced penetration of HIV into the endocervix in macaques *in vivo*. Importantly, recent work has suggested that even the type of lactobacillus bacteria present in CVM can impact HIV trapping [20], which is consistent with HIV incidence observed in South African women with different lactobacillus bacteria [12]. We hypothesize that polymicrobial vaginal microbiota communities may similarly have negative impact on CVM barrier properties to HIV, which is likely to contribute to the increased risks of acquisition and transmission of HIV in women.

Here, we investigate the mobility of HIV-1 virions and various sized nanoparticles in CVM from women with BV compared to CVM from women with *Lactobacillus*-dominant microbiota. We include CVM samples from women with polymicrobial vaginal microbiota which includes intermediate (4–6) and BV (7–10) Nugent scores, CVM samples from women with symptomatic and asymptomatic BV, and a small number of CVM samples collected before and after antibiotic treatment. Our results suggest that the resident vaginal microbiota can alter their surrounding mucin network, potentially compromising protective barrier functions in the context of infection.

## Results

### Polymicrobial and *Lactobacillus iners*-dominant vaginal microbiota are associated with reduced CVM barrier properties to HIV-1

Odeblad demonstrated that fluid is reabsorbed from mucoid substances secreted from the mucus-producing columnar cells of the endocervix as they travel through the vaginal canal [26]. The resulting mixture of mucins, shed epithelial cells, and microbiota is referred to as cervicovaginal mucus (CVM), reflecting the origination from the cervix before entering the vagina. CVM from women with *Lactobacillus crispatus*-dominant vaginal microbiota adhesively traps (immobilizes) HIV-1 [20]. A follow up study that used a modified self-collection protocol reported that CVM hindered the diffusion of HIV-1, but did not effectively trap virions [23]. To address the difference in collection methods, we did a small pilot study (n = 4) to compare the original method for quick insertion and removal of the menstrual cup [27] (<30 s) to the modified method in which the cup was inserted under the cervix for 2 h (see supporting information methods). We observed that for a given individual, leaving the cup in place for 2 h resulted in increased mucus pH and increased water content compared to quick insertion (S1 Table). While the CVM sample volumes collected were similar with both methods, the CVM samples collected using the 2 h insertion method had a runny, watery layer on top (S1A Fig). This suggests that the quick insertion approach may facilitate more accurate measurements of vaginal pH, lactic acid concentrations, and cytokine concentrations. Further, viral diffusion, as characterized by the distribution of individual virion log-transformed mean square displacements at a timescale of 1 s (log_10_(MSD(τ = 1 s)/μm^2^), was increased in mucus that was collected using the longer collection method compared to the quick insertion time (S1 Fig). Two out of the four CVM samples collected using the quick insertion method effectively trapped HIV virions (S1B Fig), whereas none of the mucus samples collected using the 2 h insertion completely trapped the virions (S1C Fig). Similarly, polystyrene microparticles (~1 μm in size) coated with PEG (PS-PEG) to reduce adhesive interactions with mucus were increasingly mobile in the CVM collected with the 2 h cup insertion time compared to the quick insertion time, reflecting the increased dilution of the mucus mesh (S2 Fig) [21]. There was a similar minor increase in the mobility of the uncoated, mucoadhesive 1 μm microparticles (PS) with the 2 h cup insertion time, suggesting a decrease in CVM adhesivity to microparticles (S2 Fig). Thus, we proceeded using the shorter cup insertion time for CVM self-collection, as CVM collected using this approach more likely reflects native CVM.

Participant demographics can be found in Table 1. The protocols and inclusion criteria and a detailed description of the numbers of samples included in each sub-study are described in the Materials and Methods. For all participant CVM samples, we grouped samples by Nugent scoring. *Lactobacillu*s-dominant samples score in the range of 0–3, intermediate is defined as scores of 4–6, and BV is defined as scores of 7–10 [28]. The Nugent 0–3 group was further divided into two subgroups based on the concentrations of D-lactic acid (D-LA) measured in CVM, as relative production of D-LA has been demonstrated to differentiate the two most common *Lactobacillus* species present in CVM, *L*. *crispatus* (high D-LA production) and *L*. *iners* (low D-LA production) [29]. Group 1 included Nugent 0–3 CVM samples with high D-LA (>0.4% w/v, S3 Fig), Group 2 included Nugent 0–3 CVM samples with low D-LA (<0.4% w/v, S3 Fig), Group 3 included Nugent 4–6 CVM samples, and Group 4 included Nugent 7–10 CVM samples (Table 2). The average D-LA and L-LA content for each Group is shown in Table 2, and the individual values for each CVM sample can be found in S3 Fig. The average sample pH was reflective of the total (D+L) LA concentrations measured, with the lowest average pH in Group 1 (3.81 ± 0.21), followed by Group 2 with a slightly higher average pH 4.22 ± 0.65. The average pH was much higher for Group 3 (5.06 ± 0.41) and Group 4 (5.08 ± 0.48) (Table 2), reflecting the relative depletion of lactic acid producing bacteria. The individual sample pH values are shown in S4 Fig.

**Table 1:** Participant demographics collected by self-report.

Age	Group 1	Group 2	Group 3	Group 4
Median (range)	25 (21–38)	27 (19–44)	28 (22–44)	27 (18–46)
**Ethnicity**				
Not Hispanic or Latino	23 (82%)	14 (74%)	9 (82%)	39 (89%)
Hispanic or Latino	3 (11%)	4 (21%)	2 (18%)	2 (4%)
Unavailable*	2 (7%)	1 (5%)	0 (0%)	3 (7%)
**Race**				
White	17 (61%)	9 (48%)	3 (27%)	10 (22%)
Black or African American	1 (3.5%)	8 (42%)	8 (73%)	29 (65%)
Asian	6 (21%)	0 (0%)	0 (0%)	0 (0%)
American Indian or Alaska Native	1 (3.5%)	0 (0%)	0 (0%)	1 (2%)
Other	1 (3.5%)	1 (5%)	0 (0%)	2 (4%)
Unavailable*	2 (7.5%)	1 (5%)	0 (0%)	3 (7%)
**Type of Birth Control**				
None	18 (64%)	10 (53%)	9 (82%)	31 (69%)
Pill Vaginal ring	3 (11%) 1 (4%)	4 (21%) 0 (0%)	0 (0%) 1 (9%)	6 (14%) 1 (2%)
Injectable	0 (0%)	1 (5%)	1 (9%)	2 (4%)
Implant	0 (0%)	0 (0%)	0 (0%)	1 (2%)
Hormonal IUD	4 (14%)	3 (16%)	0 (0%)	0 (0%)
Copper IUD	0 (0%)	0 (0%)	0 (0%)	1 (2%)
Unavailable*	2 (7%)	1 (5%)	0 (0%)	3 (7%)
**History of conditions**				
Unavailable*	2 (7%)	5 (26%)	4 (36%)	19 (42%)
None^#^	22 (85%)	9 (64%)	3 (43%)	13 (50%)
Yes, specified^#^:	4 (15%)	5 (36%)	4 (57%)	13 (50%)
Herpes	3	1	2	2
Gonorrhea	0	2	1	3
Chlamydia	3	4	3	5
Bacterial vaginosis	1	4	3	10
Yeast infection	0	0	1	2
Trichomoniasis	0	0	0	1
Human papilloma virus	0	1	1	1

*Unavailable responses were due to either blank answers, missing questionnaires, or questionnaires that did not include the question. Each participant may have self-reported diagnosis with more than one reproductive tract infection/condition, so the results are not additive. A few participants provided multiple samples over a course of weeks or months, over which time age and birth control status changed, and thus their demographics were tabulated and included for each visit. The few instances where the same participant provided multiple samples are detailed for each set of experiments in the methods.

^#^Due to large number of participants with unavailable information, percentages were calculated based only on participants where information was available.

**Table 2:** CVM sample group categorization and biochemical properties.

Group	Nugent score	D-Lactic acid (% w/v)	L-Lactic acid (%w/v)	pH
1	0–3	0.68 ± 0.23 (high)	0.46 ± 0.13	3.81 ± 0.21
2	0–3	0.20 ± 0.11 (low)	0.65 ± 0.29	4.22 ± 0.65
3	4–6	0.11 ± 0.13	0.24 ± 0.20	5.06 ± 0.41
4	7–10	0.12 ± 0.15	0.22 ± 0.15	5.08 ± 0.48

Group assignments based on Nugent score, D- and L-lactic acid content and pH. pH and lactic acid values are reported as mean ± SD.

**Fig 1:**
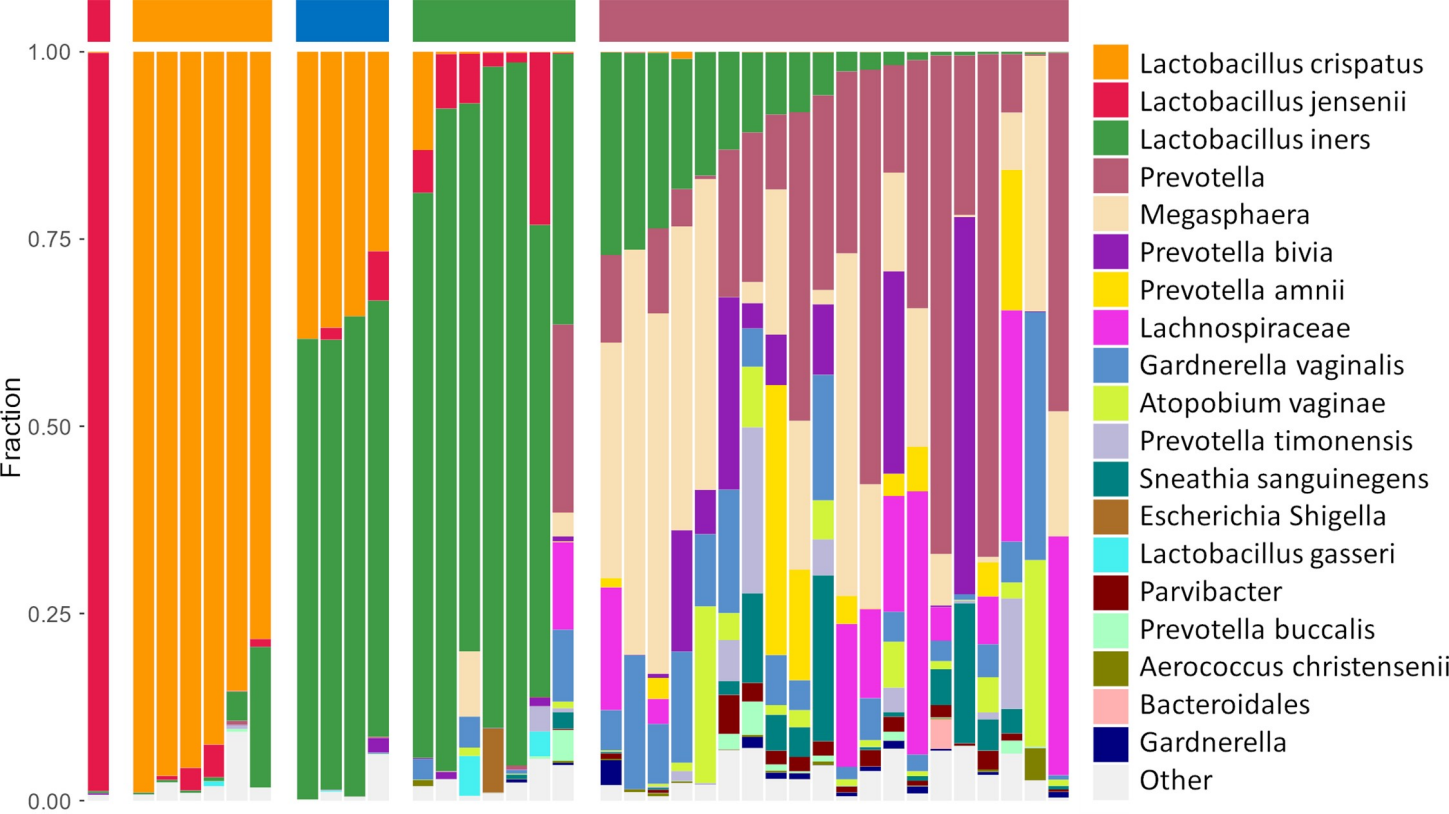
Stacked bar graph of bacterial phylotypes as determined by 16S rDNA sequencing. Samples are organized according to community state types (CST) as indicated by the colored bar on top of the graph (left to right: red = *L*. *jensenii*; orange = *L*. *crispatus*; blue = mixture of *L*. *iners* and *L*. *crispatus*; green = *L*. *iners*; maroon = polymicrobial). Each column represents an individual sample (n = 38 total). Bacterial phylotypes are indicated by different colors displayed in the legend on the right.

We next used 16S rDNA sequencing on a subset of the samples (38/84, 45%) to determine how the Group categorizations shown in Table 2 associated with the microbiota present in the samples. As shown in Fig 1, a total of 5 clusters were identified. Four clusters were *Lactobacillus*-dominant microbiota, including *L*. *crispatus* (orange bar, n = 6), *L*. *iners* (green bar, n = 7), *L*. *jensenii* (red bar, n = 1), and a mixture of *L*. *iners* and *L*. *crispatus* (blue bar, n = 4) (Fig 1). The polymicrobial group consisted of anaerobes of genus *Megasphaera*, *Prevotella*, *Gardnerella*, *Atopobium*, and others, including *L*. *iners* (maroon bar, n = 20) (Fig 1). Out of the 9 sequenced samples categorized as Group 1, 6/9 (67%) samples were clustered as *L*. *crispatus*-dominant, 2/9 (22%) clustered as *L*. *iners*/*L*. *crispatus* mixtures, and 1/9 (11%) clustered as *L*. *jensenii*-dominant. The average pH and D-LA content of the sequenced samples in Group 1 was 3.85 ± 0.25 and 0.67 ± 0.18% (w/v), respectively, which were very similar to the overall Group 1 averages (Table 2). Out of the 8 sequenced samples categorized as Group 2, 6/8 (75%) samples clustered as *L*. *iners*-dominant, while 2/8 (25%) clustered as *L*. *iners*/*L*. *crispatus* mixtures. The average pH of the sequenced samples in Group 2 was 4.39 ± 0.89, which was slightly higher than the overall Group 2 average, and the average D-LA content (0.20 ± 0.10% (w/v)) was very similar to the overall Group 2 average (Table 2). Out of the 4 sequenced samples categorized as Group 3, 3/4 (75%) samples clustered in the polymicrobial group, and 1/4 (25%) clustered as *L*. *iners*-dominant, despite containing >50% relative abundance of other bacteria, including *Prevotella*, *Megasphaera*, and *Gardnerella* (last column in *L*. *iners* cluster, Fig 1). Out of the 17 sequenced samples categorized as Group 4, 17/17 (100%) samples clustered with the polymicrobial group. Based on the data here, the sensitivity of using Nugent scoring to determine BV (Nugent 7–10, Group 4) compared to clustering in the polymicrobial group by 16S sequencing was 85% (S2 Table). The specificity, positive predictive value, and negative predictive value were 100%, 100%, and 86%, respectively (S2 Table). Consistent with prior reports, women in Group 1 were more likely to be White (61%) or Asian (21%), whereas women in Groups 3 and 4 were more likely to be Black or African American (73% and 65%, respectively). Women in Group 1 were the least likely to self-report a history of any of the conditions listed in Table 1, and birth control use was similar across all groups.

**Fig 2:**
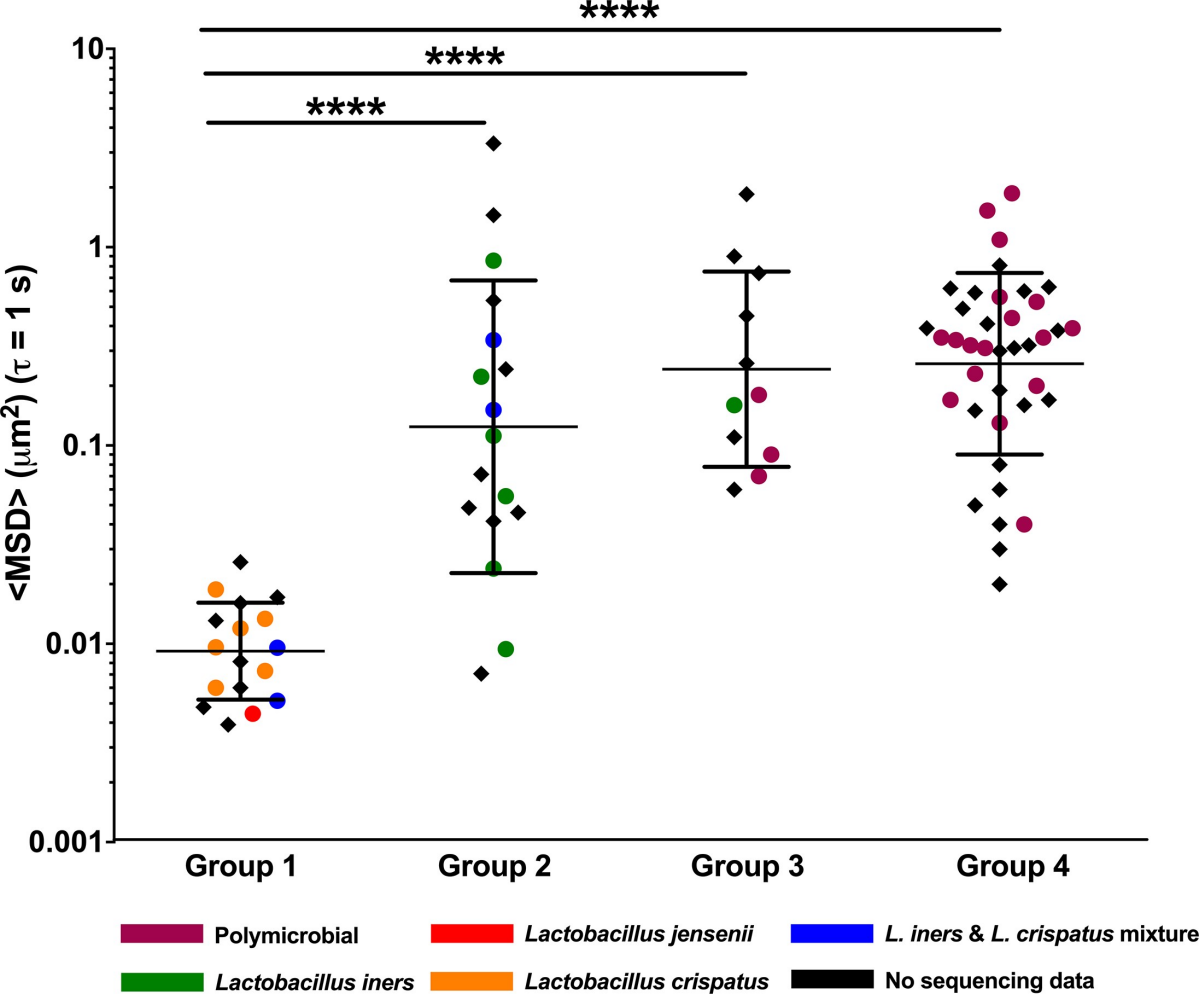
HIV virion ensemble-averaged mean square displacement <MSD> at a time scale (*τ*) of 1 s, where each data marker represents an individual CVM sample (n = 84 CVM samples from 70 participants). Samples are organized by group assignments and color coded based on available 16S sequencing community state types. Data represented as geometric mean and geometric mean standard deviation. **** p < 0.0001. Repeat samples from the same participant were excluded in statistical analyses.

Fig 2 shows the diffusional mobility of HIV-1 virions for all CVM samples, and color-coding of individual data points indicates which samples were sequenced and clustered as described above. Viral diffusion was represented as the geometric mean ensemble-averaged mean square displacement (<MSD>) at a time scale (*τ*) of 1 s, with each data point representing an individual CVM sample. A total of n = 84 samples were characterized, representing n = 70 individual women, where n = 10 of these women provided multiple samples on different days. Consistent with what we have observed previously [19, 20], CVM samples in Group 1 (17 samples from 17 women) effectively constrained the viral motion, referred to as “trapping” (Fig 2). In contrast, HIV mobility in Group 2 samples (17 samples from 16 women) was significantly higher on average, representing a ~14-fold increase in (geometric mean) <MSD> compared to Group 1, and there was higher inter-sample variability (Fig 2). For CVM samples in Group 3 (11 samples from 10 women) and Group 4 (39 samples from 34 women), HIV mobility was more uniformly increased compared to Group 1. The geometric mean <MSD> for Group 3 and Group 4 were ~26- and 28-fold higher compared to Group 1, respectively (Fig 2). The viral mobility in the few samples that clustered differently based on sequencing within each Group were particularly interesting. The single *L*. *jensenii*-dominant CVM sample in Group 1 effectively trapped HIV similar to *L*. *crispatus*-dominant samples (red dot in Group 1, Fig 2). The four samples that clustered as *L*. *iners*/*L*. *crispatus* mixtures (blue dots in Fig 2) had similar relative abundance of each species (Fig 1), but the viral mobility stratified by sample pH (Group 1 pH 3.86 and 3.87, Group 2 pH 4.29 and 4.40). Of note, mixed *L*. *iners*/*L*. *crispatus* samples are conventionally collapsed into *L*. *iners-* or *L*. *crispatus-*dominant CSTs [30], but the functional differences we observed here despite similar relative abundances of *L*. *iners* and *L*. *crispatus* led us to keep the cluster distinct. Viral mobility was similar whether participants were or were not using birth control (S5 Fig).

### Reduced CVM barrier function to HIV is not related to mucus pore structure

**Fig 3:**
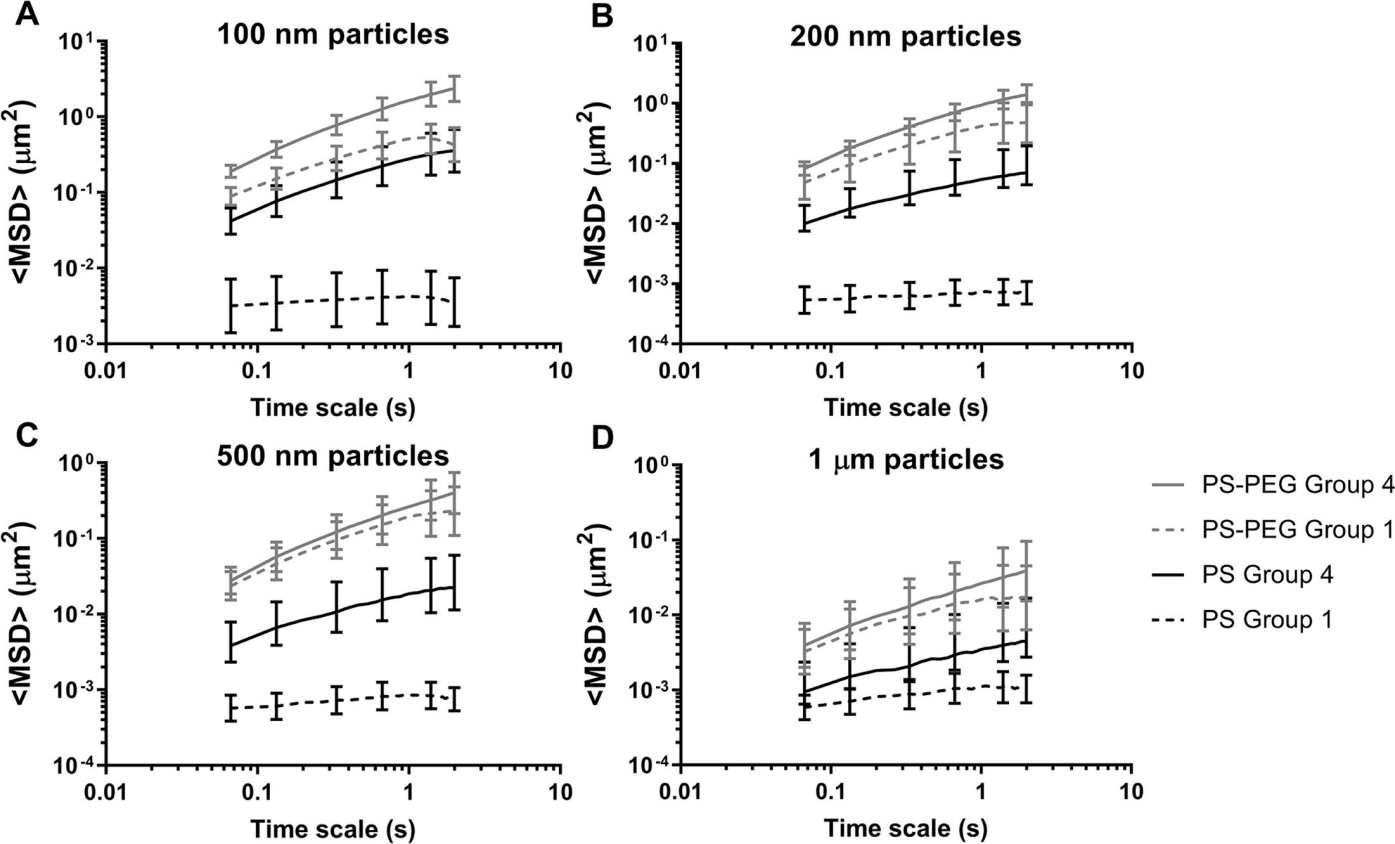
Ensemble-averaged mean square displacement (<MSD>) as a function of time scale (*τ*) for (A) 100 nm, (B) 200 nm, (C) 500 nm, and (D) 1 μm sized PS and PS-PEG particles in Group 1 (n = 6) and Group 4 (n = 8) CVM samples. Data represented as the average ± SEM.

**Fig 4:**
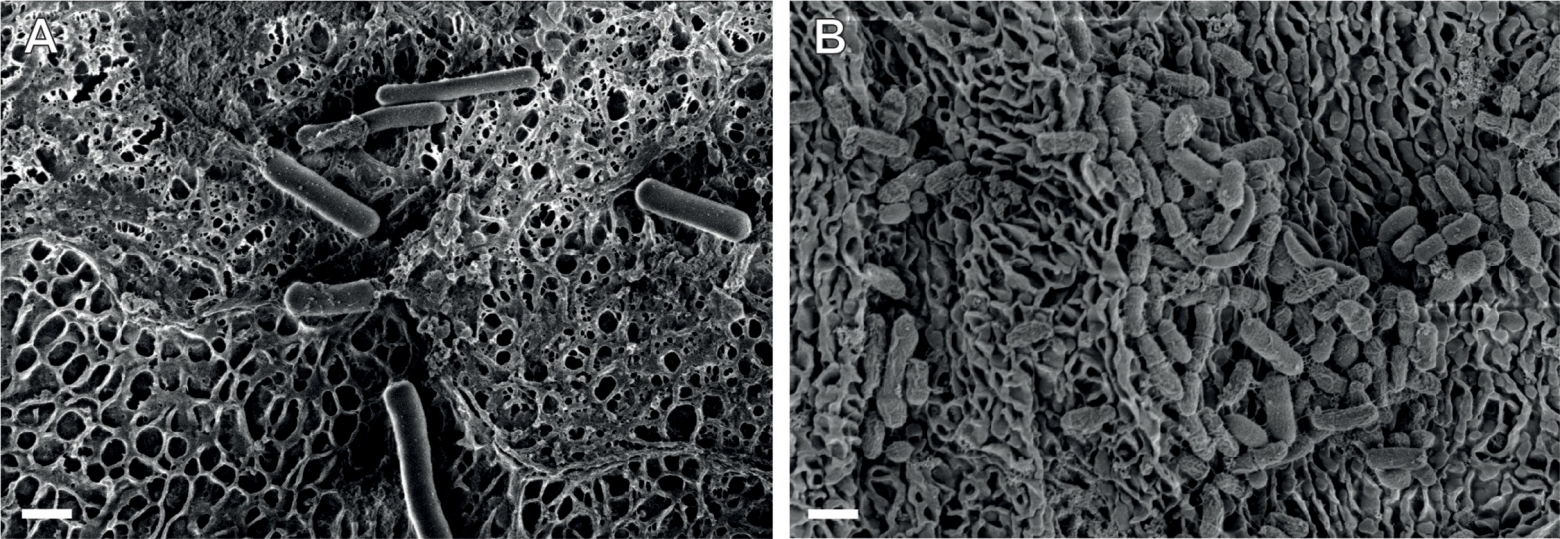
Representative scanning electron microscopy (SEM) images of (A) Group 1 and (B) Group 4 CVM samples. Scale bar = 1 μm.

We have demonstrated previously that the mechanism by which acidic, *L*. *crispatus*-dominant CVM samples trap HIV virions is by adhesive interactions with the mucus mesh rather than physical entrapment within the mucus pores (steric obstruction). This conclusion was supported by parallel observations of the mobility of various sizes of non-adhesive PS-PEG nanoparticles and HIV virions; PS-PEG nanoparticles much larger than the size of typical viruses diffused rapidly in CVM samples where HIV virions and uncoated PS nanoparticles were immobilized [20, 31]. Similarly, larger microparticles typically experience some steric obstruction in CVM, and increased mobility would be a potential measure of breakdown of the mucus pore structure [31–33]. Thus, we used various sizes (100 nm– 1 μm) of PS and PS-PEG particles to characterize mucus structure in BV CVM samples (Group 4) compared to *L*. *crispatus*-dominant CVM samples (Group 1). As shown in Fig 3, there were no statistically significant differences in the mobility of any sized (non-adhesive) PS-PEG particles. In contrast, there were clear differences in the mobility of PS particles of every size (statistically significant by t-test except for 1 μm PS), which showed increased mobility in Group 4 compared to Group 1 CVM samples (Fig 3). Thus, the increased mobility of both HIV virions and PS particles reflects decreased adhesivity in BV CVM, while little change in PS-PEG mobility suggests intact physical pore microstructure. To confirm this, we used scanning electron microscopy (SEM) to visualize the CVM microstructure. We imaged n = 3 CVM samples from Group 1 and n = 3 from Group 4. Representative images are shown in Fig 4, while additional images from the other CVM samples are shown in S6 Fig. Group 1 samples contained a heterogeneous pore microstructure, consistent with our prior observations of a wide distribution of pore sizes using particle tracking [21], with large lactobacillus rods interspersed (Fig 4A). BV CVM also contained networks of intact pores (Fig 4B), consistent with the PS-PEG particle tracking data (Fig 3). The apparent high amounts of bacteria and biofilm present may actually be additive to the physical barrier properties in some BV CVM samples (S6 Fig).

### CVM adhesive barrier properties are decreased in BV whether symptomatic or asymptomatic

**Fig 5:**
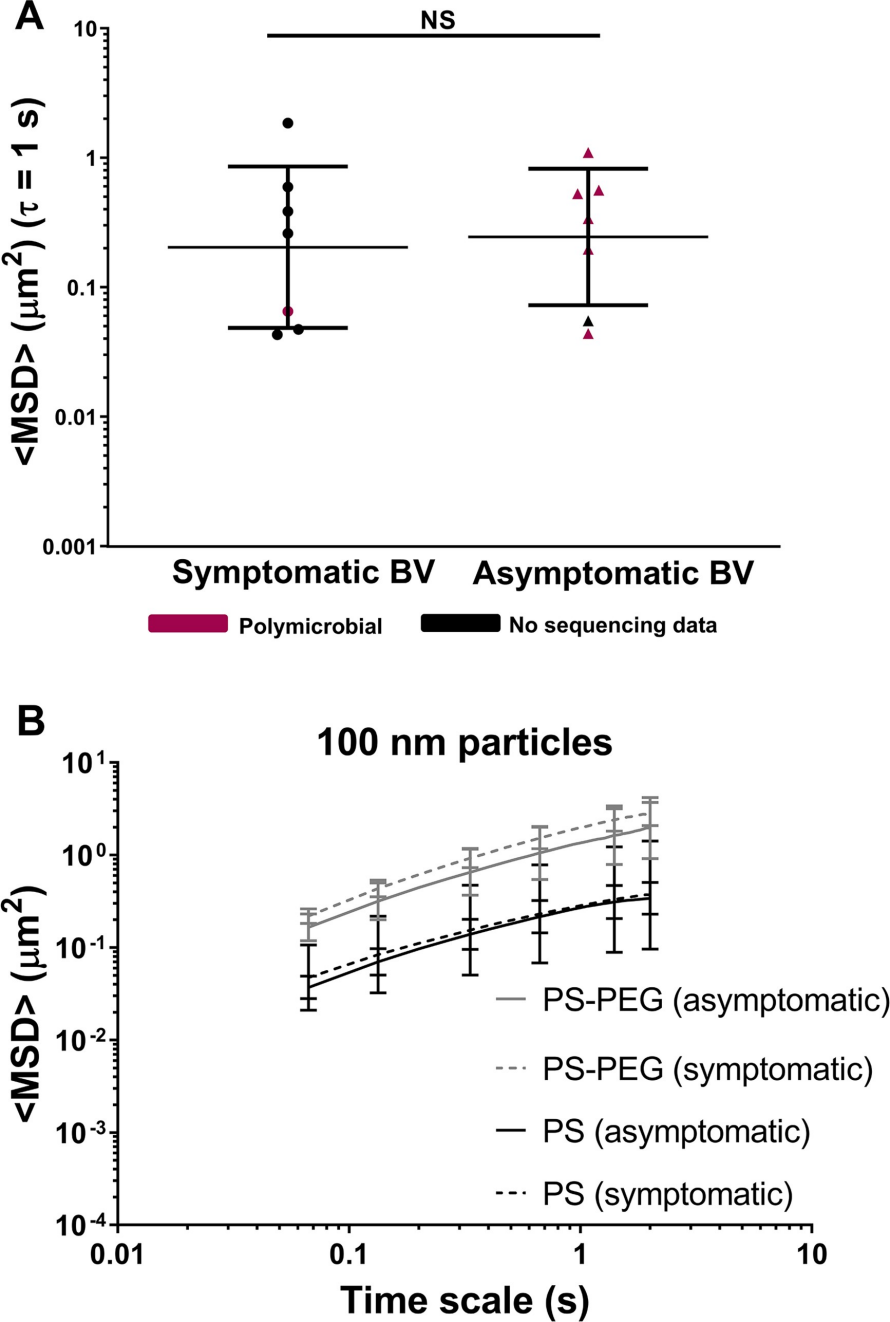
(A) HIV virion ensemble-averaged mean square displacement <MSD> at a time scale (*τ*) of 1 s in CVM from participants with symptomatic BV (n = 7) compared to asymptomatic BV (n = 7). Data is redundant from selected samples also shown in Fig 2. Samples are color-coded based on available 16S sequencing community state types. Data represented as geometric mean and geometric mean standard deviation. (B) Ensemble-averaged mean square displacement <MSD> as a function of *τ* for 100 nm PS and PS-PEG particles in CVM samples from participants with symptomatic BV (n = 4) compared to asymptomatic BV (n = 4). Data is redundant from selected samples also shown in Fig 3. Data represented as average ± SEM.

A small subset of participants were patients seeking treatment for symptomatic BV. We thus compared the CVM samples obtained from these women to a random subset of women whose CVM samples were only identified as BV in the laboratory (asymptomatic). As shown in Fig 5A, we found no significant difference in HIV mobility between symptomatic and asymptomatic BV (n = 7 each). Further, the mobility of non-adhesive 100 nm PS-PEG particles, similar in size to HIV virus, was similar in CVM whether BV was symptomatic or asymptomatic (n = 4 each), and reflective of unobstructed diffusion (Fig 5B). Indeed, there was also a marked increase in the mobility of 100 nm PS nanoparticles (Fig 5B), which are typically trapped by adhesive interactions in Group 1 CVM samples (Fig 3A), regardless of whether BV was symptomatic or asymptomatic. Together, these results suggest that the reduced CVM adhesive barrier properties in BV are present regardless of whether women perceive symptoms that are significant enough to seek treatment. Further, a few women with symptomatic BV (n = 4) provided a follow-up CVM sample 4–6 weeks after completing antibiotic treatment for BV. As shown in S7 Fig, the <MSD> for HIV virions in each participant’s CVM samples did not differ significantly before and after antibiotic treatment, and reflected high viral mobility compared to the adhesive trapping in Group 1 samples (dotted line). This is consistent with the increased viral mobility observed in both Group 4 samples and most Group 2 CVM samples shown in Fig 2.

### HIV mobility correlates with pH and lactic acid content

**Fig 6:**
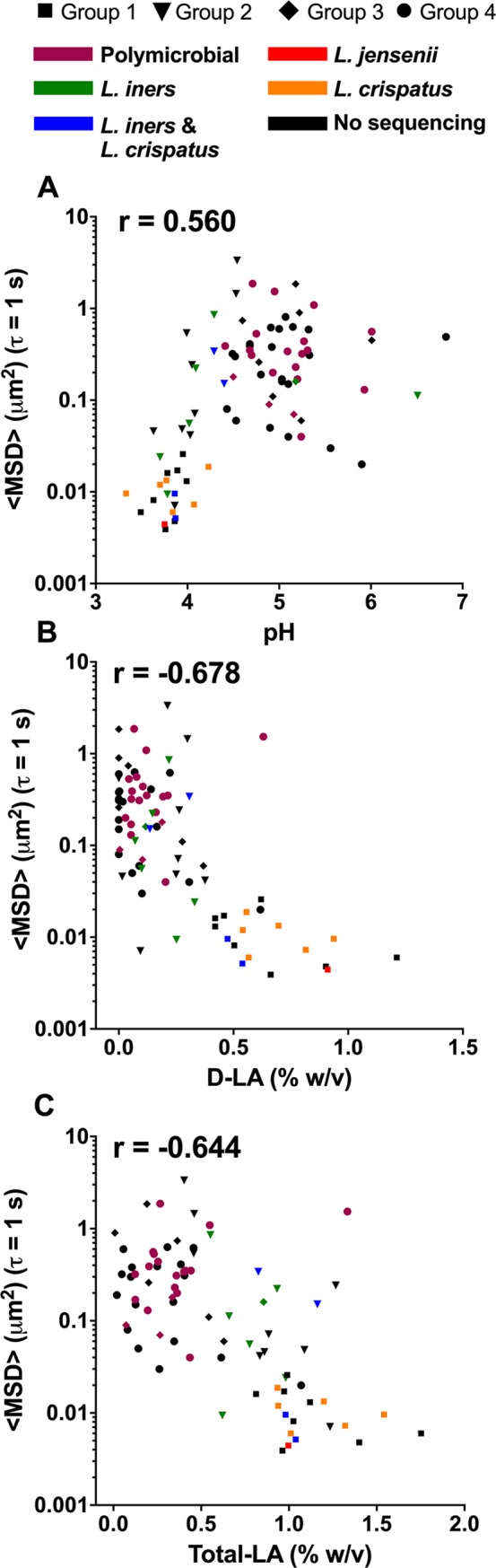
HIV virion ensemble-averaged mean square displacement <MSD> at a time scale (*τ*) of 1 s in CVM plotted as a function of (A) pH (n = 84 CVM samples) (B) D-LA (n = 79 CVM samples) and (C) total lactic acid (n = 79 CVM samples). The shape of the data marker reflects the group assignment and the color reflects community state types based on available 16S sequencing data. Pearson correlation coefficient (r) value shown on graph. All p values were p<0.0001.

As pH is a key diagnostic criterion for BV, we observed how <MSD> values varied with the pH of each CVM sample. For all samples in Groups 1–4, we observed a positive correlation (r = 0.56, p<0.0001) with HIV mobility increasing with pH (Fig 6A). Of note, the three samples in Group 2 with the lowest HIV mobility (Fig 2) also had lower pH similar to the Group 1 samples (Fig 6A). Also, as described previously, the two CVM samples clustering in the sequencing as *L*. *iners/L*. *crispatus* mixtures and categorized as Group 1 (Fig 2, trapped HIV) had lower pH (Fig 6A, blue squares), whereas the two samples in Group 2 (Fig 2, high HIV mobility) had higher pH (Fig 6A, blue triangles). The acidic pH of CVM is caused by lactic acid, so we also examined whether HIV mobility varies as a function of concentrations of D-LA and total LA. We observed a strong negative correlation between <MSD> and D-LA concentrations (r = -0.678; p<0.0001) (Fig 6B) and between <MSD> and total lactic acid (r = -0.644; p<0.0001) (Fig 6C). Notably, the four *L*. *iners/L*. *crispatus* mixture CVM samples had similar total lactic acid concentrations (Fig 6C), but the two samples that trapped HIV and were categorized as Group 1 had higher D-LA content (Fig 6B, blue squares), while the two samples that did not trap HIV had lower D-LA content (Fig 6B, blue triangles) and were categorized with Group 2.

## Discussion

An important feature of any mucosal surface that provides protection against infection is the mucus barrier [34–36]. Secreted mucus serves as the first line of defense through steric and adhesive immobilization of pathogens and removal through mucus clearance [35]. Vaginal microbiota also live within the cervicovaginal mucus (CVM), and thus the composition of vaginal microbiota can have a direct impact on the structure and function of CVM. Here, we characterized the HIV barrier properties (Fig 2) and the structural properties (Figs 3 and 4) of CVM from women with *Lactobacillus*-dominant microbiota as well as those with polymicrobial microbiota. Importantly, we separately assessed women with asymptomatic and intermediate BV, as these women are typically not included in studies for understanding disease risks. Further, given the recurrent nature of BV, we included women before and after antibiotic treatment. Consistently, we demonstrated that the native diffusional barrier properties of CVM against HIV are reduced in women with *L*. *iners*-dominant and polymicrobial microbiota. Only CVM from Group 1, which had high D-LA concentration and low pH reflective of predominantly *L*. *crispatus-*dominant microbiota consistently trapped HIV.

CVM is essentially a hydrogel composed of mucins that form a porous net structure with viscoelastic properties. Mucins further help support and maintain mucosal microbiota by providing binding sites for commensal bacteria and glycans as a nutrient source for certain bacteria [37]. Clinically, symptoms of BV often include descriptions of discharge that is thin or watery, which would be associated with decreased viscoelasticity. Indeed, it was reported that cervicovaginal lavage fluid from women with BV had the lowest viscosity measured by rheometry, even when counterbalanced by the increased viscosity that has been described with use of hormonal contraceptives [38]. However, when characterizing the undiluted CVM from women with BV, we rarely observed markedly reduced viscoelastic properties microscopically by particle tracking. With SEM, we observed a network of mucin bundles with heterogeneous pore sizes from both women with (Fig 4B) and without BV (Fig 4A). Further, CVM samples from women with BV suggested there may even be increased microscopic density due to high amounts of bacteria and biofilm. Our prior work using multiple particle tracking to probe the microstructure of *Lactobacillus*-dominant CVM also reflected heterogeneous pore sizes ranging from ~50 nm to >750 nm [21]. Considering HIV is ~120 nm in diameter, we previously concluded that HIV virions were trapped due to adhesive interactions rather than steric hindrance [19, 39]. Similarly, the adhesive and non-adhesive nanoparticle mobility in BV CVM summarized here supports the idea that HIV virions have increased mobility in BV CVM due to reduced adhesive interactions with the mucin meshwork.

About half of women with BV are asymptomatic [40, 41]. When comparing asymptomatic and symptomatic BV, the mobility of muco-inert particles (PS-PEG) suggested the CVM microstructure was largely intact and not significantly different (Fig 5). Further, adhesive nanoparticles (PS) and HIV virions were similarly mobile in CVM from women with symptomatic and asymptomatic BV, suggesting similar diminished HIV barrier function. Moreover, women with BV had elevated levels of genital pro-inflammatory cytokines regardless of BV symptoms [40]. CDC guidelines recommend treatment only for symptomatic cases of BV [42], though evidence is accumulating that suggests women with asymptomatic BV may be at similar elevated risk for HIV infection as women with symptomatic BV [43]. However, as discussed above, standard antibiotic treatment does not result in the restoration of an optimal and protective vaginal microbiota. Thus, it is unclear whether using standard treatment approaches for asymptomatic BV would provide any benefit.

In research settings, Nugent scores remains the gold standard for BV diagnosis [41]. Furthermore, Nugent scores are often used for participant screening and inclusion/exclusion criteria in clinical studies. However, Nugent scoring is relatively subjective and requires a well-trained microscopist. Here, we showed that our Nugent scoring was sensitive (85%) and specific (100%) for identifying polymicrobial bacteria communities with 100% positive predictive value compared to 16S rDNA sequencing (S2 Table). If we included the samples with intermediate Nugent scores (4–6), which typically contain a significant proportion of non-lactobacillus bacteria, the sensitivity of the Nugent scoring would have increased to 95%. When considering CVM barrier properties to HIV virions, Nugent 4–6 (Group 3) samples were indistinguishable from Nugent 7–10 (Group 4) samples. Generally, studies have either excluded participants with intermediate Nugent scores or, more commonly, grouped intermediate together with Nugent scores 0–3, classifying them as BV negative participants [4]. Our results suggest that merging these two groups may significantly underestimate the deleterious effects of BV.

BV and BV-associated bacteria have been shown to be associated with elevated levels of mucin degradative enzymes such as sialidase, glycosidase, mucinase, prolidase etc. [44–47]. Lewis et al. previously demonstrated that *Gardnerella vaginalis* produces sialidase that can be used to recapitulate mucus degradation in a murine model [48–50]. Chappell et al. similarly hypothesized that mucus degrading enzymes in BV may reduce the barrier function of mucus to HIV [38]. Here, we directly report that CVM from women with BV/polymicrobial communities have reduced adhesivity to HIV virions, allowing for increased viral mobility. It is possible that the reduction in adhesive interactions could be related to elevated levels of mucin degrading enzymes, which is an area of ongoing investigation in our lab. Explorations into how polymicrobial vaginal microbiota communities modulate the mucus barrier may provide insight into adjuvant therapies for HIV/STI prevention.

The lactic acid produced by *Lactobacillus* spp. may also provide protection against HIV infection by inactivating both cell-associated and cell-free HIV [51, 52]. Undiluted CVM at low pH (<pH 4) has potent *ex vivo* anti-HIV-1 activity due to protonated lactic acid [53]. Lactic acid was also shown to have immunomodulatory effects in cervicovaginal epithelial cell lines, including increased production of anti-inflammatory cytokine IL-1RA, reduction in pro-inflammatory cytokines, and dampened response to Toll-like receptor induced inflammation [54]. Here, we found that higher levels of D-LA and total LA were associated with decreased HIV mobility in CVM. However, it was previously reported that the addition of lactic acid to CVM with reduced adhesive properties did not restore HIV adhesion [20]. Thus, the concentrations of lactic acid were an indicator of the composition of the vaginal microbiota, but did not have a direct effect on viral adhesion. Similarly, the observation here that increased pH was associated with increased viral mobility is confounded by the fact that pH is also related to the composition of the vaginal microbiota. Indeed, a recent report by O'Hanlon et al. showed that *in vivo* vaginal pH strongly correlated to lactate concentrations [55].

The relatively low probability of male-to-female HIV transmission per coital act (0.08% in high income countries and 0.3% in low income countries) suggests that the innate barrier function of the genital mucosa is robust [56]. There are some limitations to using fresh, undiluted CVM for studying viral interactions, as we are unable to mimic neutralization and dilution by semen. Stirring/mixing CVM and semen together would not mimic the complex nature of the fluid interface and the pH gradient that likely occurs in vivo [20, 57]. Masters and Johnson previously demonstrated that vaginal pH measured with an insertive electrode rapidly increases upon ejaculation and recovers within ~2 h with the ejaculate remaining in the vagina, which is consistent with the measured rate of lactic acid production by bacteria in culture [58, 59]. Unfortunately, the rates of semen clearance due to gravity, ambulation, or cleansing practices is not well understood, but would likely affect the rate at which vaginal acidity is restored. Investigating the effect of semen exposure on CVM barrier function *in vivo* will be critical to fully understanding the impact on HIV acquisition risk. Another limitation of the data reported here is that we are unable to address how CVM may act as a barrier to cell-associated HIV, though the relative contribution of cell-free and cell-associated HIV in sexual transmission is not well understood [60]. The samples collected in this study also excluded the potential impact of menses and ovulation, and we did not determine whether participants not using hormonal contraception were in the luteal or follicular phase, all of which could affect CVM barrier function. In the context of female-to-male transmission, the innate barrier function of *Lactobacillus*-dominant CVM alone may play a role, as virus shed into the vaginal fluids would be immobilized and inactivated. Indeed, a 3-fold increased risk of female-to-male HIV transmission was observed in women with BV [13], which does not seem to be due to increased vaginal HIV RNA shedding [61]. HIV must also be able to transverse the mucus to reach the inner foreskin and glans epithelia sites of transmission [62].

In summary, we demonstrated that the CVM of women with polymicrobial vaginal microbiota is more permissive to HIV virions, which is in agreement with published associations between BV and increased risk for HIV acquisition and transmission. A similar average increase in HIV mobility with high inter-sample variability was observed in *Lactobacillus iners*-dominant CVM. Further, the observed alterations in mobility of HIV and nanoparticles was similar regardless of presence or absence of symptoms of BV. Innovation is desperately needed to identify the next generation of treatment and prevention approaches for BV.

## Materials and methods

### Ethics statement

Samples were collected as part of several ongoing studies aimed at characterizing the physicochemical properties of cervicovaginal mucus (CVM) over the period of 2013–2019. Protocols and procedures for CVM/swab self-collection were approved by the Johns Hopkins University Institutional Review Boards under IRB studies NA_00038105, NA_00085130 and HIRB00000526. Written informed consent was obtained from all human subjects prior to participation. All participants were adults between the ages of 18–46 years old.

### Participant demographics

The studies described here were conducted as part of several ongoing investigations and were observational in nature rather than prospective. Participants in study NA_00038105 were largely recruited at the Johns Hopkins School of Medicine East Baltimore campus using posted flyers and word of mouth. Study NA_00085130 included participants recruited at the East Baltimore campus (including the Johns Hopkins Outpatient Center) and the Johns Hopkins Bayview campus. Study HIRB00000526 included participants recruited at the Johns Hopkins Homewood campus and the Druid Hill STD clinic. Each study used questionnaires to collect self-reported demographic information, reported in Table 1. The questionnaires used for participants recruited from the clinics did not ask for self-reported history of infections/conditions of the female reproductive tract, and thus was tabulated as “missing” in Table 1. A few participants provided multiple CVM samples throughout the course of the studies, and because their age and birth control status changed, their demographic information was individually tabulated for each visit. For the data set compiled in Fig 2, all CVM samples in Group 1 were provided by individual participants. For Group 2, one participant provided two CVM samples spaced out by ~1.5 years, and all other samples were from individual participants. For Group 3, one participant provided 2 CVM samples spaced 5 months apart, between which she ceased using birth control. All other Group 3 samples were from individual participants. For Group 4, one participant was originally recruited from the clinic after diagnosis for BV and provided a total of 4 CVM samples between a period spanning 2013–2016; one participant provided 3 CVM samples over a period of 6 months during which her birth control changed; one participant was originally recruited from the clinic after diagnosis for BV and provided 2 CVM samples spaced out by ~1 year; one participant provided 2 CVM samples spaced 4 months apart, between which she had a copper IUD removed. All other Group 4 samples were from individual participants. For the data set compiled in Figs 3 and 5 of 8 participants from Group 4 also provided CVM samples for HIV tracking in Fig 2. For the data set compiled in Fig 5, all data are representative of a subset of samples also shown in Fig 2 and Fig 3. The number of CVM samples was set based on availability of data for samples that fell in the symptomatic category (n = 7 for Fig 5A, n = 4 for Fig 5B), and a random set of complementary samples from the asymptomatic category was selected for comparison. For the data set compiled in S7 Fig, two of the pre-treatment data points and one of the post-treatment data points are also shown in Fig 2.

### Cervicovaginal mucus (CVM) collection and characterization

The CVM collection protocol was similar to that described previously by our group [27]. Participants were eligible if they were premenopausal and at least 18 years old. Exclusion criteria for providing CVM samples included: (i) currently on or within 3 days of the end of their menstrual period; (ii) had unprotected vaginal intercourse within the past 3 days; (iii) had protected vaginal intercourse within the last 24 h; (iv) were on antibiotics within the past 3 days; and (v) were currently ovulating as determined by urine test and observation of spinnbarkeit. Participants were instructed to insert the menstrual cup (Instead Softcup; Evofem) into their vagina for ~15 s, twist it to collect mucus from the vaginal wall during removal, and place the cup into a Falcon 50 mL conical tube. For the longer samples collection studies, the cup was left in place for 2 h prior to removal (more details in Supporting Information Methods). In some cases, participants also self-collected a vaginal swab (BD ESwab). All participants provided a urine sample for pregnancy and ovulation testing using Wondfo Pregnancy Test Strips and Clearblue digital ovulation test, respectively. CVM samples were excluded from the data herein if the participant was pregnant. CVM samples were centrifuged at 1000 rcf for 2 min to collect undiluted mucus. CVM was transferred to an Eppendorf tube using a 50 μL Wiretrol (Drummond Scientific), stored at 4 ˚C during experimentation, and fully characterized within 24 h of collection.

Clinical BV diagnoses are determined based on satisfying 3 out of 4 Amsel’s criteria: (i) thin and homogeneous discharge, (ii) pH >4.5, (iii) fishy odor upon mixing with 10% KOH, and (iv) presence of clue cells in the wet mount [63]. For all CVM samples, we are able to directly characterize criteria (ii)-(iv) in the laboratory. pH was measured using a Mettler Toledo EL20 pH meter with a micro-combination pH electrode MI-411 (Microelectrodes, Inc.). Slides were prepared for wet mount by rolling a swab coated in secretions on a standard microscope slide followed by the addition of 10 μL of normal saline and covered with a glass coverslip. The wet mount slide was observed for the presence of clue cells using differential interference contrast (DIC) microscopy. The whiff test was performed by dipping a cotton swab in the CVM and pipetting 100 μl of 10% KOH onto the swab. In research settings, BV is further categorized by gram staining and scoring a vaginal smear for different bacteria morphotypes using Nugent’s score [28]. Nugent score of 0–3 is considered “healthy”, 4–6 is intermediate, and 7–10 is considered BV. Of note, 4 of the CVM samples included in Group 4 scored in the Nugent 7–10 category and had a positive whiff test and clue cells, but the measured pH fell between 4.41–4.49. CVM samples classified as “asymptomatic” BV were self-collected by women participating in the study that self-reported being healthy and free of vaginal symptoms, but had a Nugent score in the 7–10 range. CVM samples obtained from women seeking treatment for symptoms and that received a clinical BV diagnosis in the Johns Hopkins Outpatient Center or the Druid Hill STD Clinic and directly referred to our lab for participation were considered “symptomatic” for BV. Women were only referred if they were being treated for BV without additional sexually transmitted infections. Of the women recruited in the symptomatic group, 4 of them returned to the lab 4–6 weeks after using their prescribed antibiotics (2 reported using metronidazole, 2 did not disclose the type of antibiotics prescribed) to provide a “post treatment” CVM sample. For D/L lactic acid determination, ~10 μL (5–15 mg) of CVM was transferred to an Eppendorf tube and diluted with 490 μL (if pH > 4.5) or 990 μL (if pH < 4.5) of normal saline to ensure the measurements fell within the linear range of the assay. Prior to the lactic acid assay, diluted samples were thawed and centrifuged on benchtop centrifuge at 1000 rcf for 5 minutes to pellet mucus solids. The supernatant was processed per manufacturer’s instruction using D/L-lactic acid kits (R-Biopharm).

### Nanoparticle preparation and characterization

Fluorescent carboxylate polystyrene (PS) particles 100, 200, 500 and 1000 nm (1μm) in size (FluoSpheres, Molecular Probes) were coated with polyethylene glycol (PEG) as previously described [64]. Particle size and surface charge (ζ-potential) shown in S3 Table were measured with a Malvern Zetasizer Nano ZS (173° scattering angle). Particles were diluted 1:1000 in 10 mM NaCl (pH 7) for ζ-potential measurements. PS-PEG particles had a near neutral surface charge (ζ-potential) compared to negatively charged PS particles.

### Preparation of fluorescent HIV-1 virus-like particles

Replication-defective HIV-1, internally labeled with a mCherry-Gag construct to avoid alteration of the viral surface, was prepared as previously described [20]. Briefly, plasmids encoding NL4-3Luc^+^Vpr^-^Env^-^, Gag-mCherry, and YU2 Env were transfected into 293T cells at a 4:1:1 ratio. 293T cells from UNC Tissue Culture Facility Core were sourced from ATCC. The cell supernatant was collected 48 h later, and fluorescently tagged virions from the cell supernatant were purified by centrifugation through 25% sucrose at 160,000 X g for 2.5 h. The virions were then washed, resuspended in phosphate-buffered saline (PBS), divided into aliquots, and stored at -80°C.

### Multiple particle tracking of HIV virions and nanoparticles in CVM

Fluorescently labeled HIV virions (0.3 μL) or fluorescent nanoparticles (0.5 μL) were added to 30 μL of undiluted CVM in custom-made glass slides, mixed, and immediately sealed with a glass coverslip. Stock (2.0% solids) PS and PS-PEG particles were diluted 10-400-fold with UltraPure water depending on the particle size. Twenty second videos of viral and nanoparticle diffusion in CVM were recorded at room temperature using a Zeiss Axio Observer inverted epifluorescence microscope equipped with a 100x/1.46 NA oil-immersion objective and an EM-CCD camera (Evolve 512; Photometrics) with image resolution of 25 nm/pixel and at a frame rate of 15 Hz. A minimum of 3 videos were collected per CVM sample. Nanoparticle trajectories were analyzed using automated MATLAB-based particle tracking software with a minimum of 16 frames (~1 s) of consecutive tracking as previously published [31, 65, 66]. Virions were considered “trapped” if by visual observation there was no spatial displacement during the full duration of the movie; the varying contrast and autofluorescence in the videos introduced static error that precluded the use of a standard minimum MSD value to define trapping. Representative videos of HIV-1 “trapping” in *L*. *crispatus*-dominant CVM (S1 Video) and HIV-1 diffusing in polymicrobial CVM (S2 Video) are available in the supporting information media files.

### Scanning electron microscopy (SEM)

A small aliquot (~2–5 μL) of CVM was loaded directly onto poly-L-lysine treated 12 mm diameter German round glass coverslip. The mucus was flattened with a second coverslip and the coverslips separated horizontally. The coverslips were loaded sample side up into a tissue culture plate (24-well). The samples were fixed using 1 mL solution of 2.5% w/v glutaraldehyde, 3 mM MgCl_2_, 1% sucrose in 0.1 M Sorensen’s phosphate buffer (pH 7.2). Fixation time was either 1 h at room temperature if post-fixation steps could be performed the same day, or for 24–48 h in the cold room if post-fixation steps were scheduled for a later day, according to scheduling availability with imaging facility staff. After fixative treatment, the solution was removed and rinsed 3 times for 15 min each with a solution of 0.1 M Sorensen’s phosphate buffer (pH 7.2), 3mM MgCl_2_ and 3% sucrose. Samples were then postfixed in 1% osmium tetroxide in buffer for 1 h on ice in the dark, rinsed twice with distilled water, and dehydrated with a graded ethanol series. Samples were dried with hexamethyldisilazane (HMDS) and mounted on carbon-coated stubs. Samples were sputter coated with 20 nm AuPd mixture in a Denton Vacuum Desk III Sputter Coater. The samples were imaged in a LEO/Zeiss Field-emission SEM at 1 kV.

### 16s rDNA sequencing and analysis

#### Nucleic acid extraction method

Total nucleic acid extraction was performed at the Ragon Institute according to a method adapted slightly from Anahtar et al. [67]. Samples were thawed on ice, then placed into a solution of phenol:chloroform:isoamyl alcohol (25:24:1, pH 6.6, Invitrogen), 20% sodium dodecyl sulfate (Fisher), Tris-EDTA buffer, and 0.1 mm glass beads (BioSpec). For mucus samples, 200 μl of sample (or the entirety of the sample if volume was <200 μl) was mixed into the extraction solution by vigorous pipetting, then incubated on ice. For swab samples, swabs were inserted into the extraction solution and vigorously rubbed against tube walls for 30 s to dislodge sample material from the swab, then incubated on ice for 5–10 min. Excess fluid was squeezed from the swab using pressure from a sterile pipette tip against the side of the tube and the swab was removed from the sample. Samples were homogenized for 2 minutes at 4°C using a bead beater, then centrifuged at 6,800 rcf for 3 min at 4°C. The aqueous phase was transferred to a clean tube, an equal volume of phenol:chloroform:isoamyl alcohol was added and mixed thoroughly by inversion, then samples were centrifuged at 16,000 rcf for 5 min at 4°C. The aqueous phase was removed to a clean tube, taking care to avoid any of the organic phase, then 0.8 volume of -20°C isopropanol (Sigma, molecular biology grade) and 0.08 volume (relative to initial sample) of 3M sodium acetate (pH 5.5, Life Technologies) were added. Samples were inverted to mix, then precipitated overnight at -20°C. Precipitated samples were centrifuged at 21,100 rcf for 30 min at 4°C, and the supernatant was discarded. Pellets were washed by adding 0.5 mL of 200 proof ethanol (Decon), followed by centrifugation at 21,100 rcf for 15 minutes at 4°C and removal of supernatant. The nucleic acid pellet was allowed to air dry, then was resuspended in 20 μL of molecular grade Tris-EDTA buffer. Nucleic acid concentration was measured using a Nanodrop (Thermo Scientific).

#### Amplification/library preparation method

PCR amplification of the 16S rRNA gene V4 region was performed according to method of Caporaso et al. [68]. Samples were amplified in triplicate using 0.25 μl Q5 High-Fidelity DNA Polymerase (NEB) per 25 μl reaction with 0.2 mM dNTPs (Sigma), 1X Q5 Reaction Buffer, 200 pM 515F primer (IDT), and 200 pM barcoded 806R primer (IDT) in PCR-Clean water (MoBio). A water-template negative control reaction was included with each sample to confirm absence of contaminating DNA. Samples were amplified at 98°C for 30 seconds, followed by 35 cycles of 98°C for 10 s, 60°C for 30 s, and 72°C for 20 s with a final 2 min extension at 72°C. Triplicate reactions were pooled and then separated on an agarose gel in parallel with the paired water control reactions to confirm successful amplification. Individual samples were pooled and purified using an UltraClean 96 PCR Cleanup Kit (Qiagen). The pooled library was then quantified, diluted, supplemented with 10% PhiX, chemically and heat denatured as per Illumina MiSeq protocols, and sequenced at 10 pM on an Illumina V2 1x300 sequencing kit using custom sequencing primers as per Caporaso [68].

#### Sequence processing

QIIME 1 version 1.9.1 was used to demultiplex sequences [69]. A QIIME 1 formatted mapping file was created and validated using validate_mapping_file.py, the samples were demultiplexed using split_libraries_fastq.py with parameter store_demultiplexed_fastq, then split into individual fastq files using split_sequence_file_on_sample_ids.py. The resulting sequences were then analyzed in in R using dada2 version 1.6.0 [70]. Raw sequences were filtered and trimmed at positions 10 (left) and 230 (right) using the DADA2 filterAndTrim function with parameters MaxEE = 2, truncQ = 11, and MaxN = 0. Sequences were inferred, chimeras were removed, and taxonomy was assigned using the DADA2 assign Taxonomy function employing the RDP training database rdp_train_set_16.fa.gz. Species assignments were made by exact sequence matching with DADA2 using the RDP species assignment database rdp_species_assignment_16.fa.gz (obtained from https://www.mothur.org/wiki/RDP_reference_files). Output of dada2 analysis was then analyzed in R using phyloseq version 1.22.3 [71]. Species level taxonomy assignment of ASVs assigned to the *Lactobacillus* genus was performed using BLAST and the 16S ribosomal RNA (Bacteria and Archea) database [72]. Five CSTs were assigned in R using vegan version 2.5–3 to calculate Bray-Curtis dissimilarity indices, and cluster version 2.0.7–1 to perform clustering using a partitioning around medoids method previously described [73]. The stacked barplot showing the phylotype representation in different samples was created in R using ggplot2 version 3.1.0 after sorting samples by CST.

### Statistical analysis

One-way analysis of variance (ANOVA) followed by Tukey’s multiple comparison test was used for multi-group comparisons in GraphPad Prism 7.03. Student’s t-test (two-tailed, unpaired) was used for comparison between two groups in GraphPad Prism 7.03. Statistics were done using log-transformed values of <MSD> at 1 s. Repeated samples collected longitudinally from the same participant were excluded for statistical analysis. Pearson correlation coefficient (two-tailed) was calculated in GraphPad Prism 7.03 for pH, D-LA and total LA versus log_10_ MSD values for pooled analyses. Sensitivity, specificity and positive and negative predictive values were calculated for BV diagnosis by Nugent score using 16S rDNA as the reference standard or true diagnosis.

## Supporting information

S1 Fig Distribution of individual virion log-transformed mean square displacements at a time scale (*τ*) of 1 s (log_10_(MSD((*τ* = 1 s)/μm^2^)) for quick insertion (<30s, top row) compared to 2 h insertion (bottom row) of the Softcup. Individual virion data from each sample was binned and the percentage of total virions in each bin is displayed. Data for each participant is shown individually, as there was significant variability in the fraction of virions trapped in the samples with the Quick Insert. Overall, the 2 h insertion led to an increase in virion mobility in CVM for all participants (n = 4 participants, S1-4).
(TIF)

S2 Fig Distribution of individual particle (1 μm PS-PEG and PS) log-transformed mean square displacements at time scale (*τ*) of 1 s (log_10_(MSD((*τ* = 1 s)/μm^2^)) for a quick insertion (<30s) compared to a 2 h insertion of the Softcup. Individual particle data from each sample was binned, and the percentage of total particles in each bin was averaged over n = 3 participants.
(TIF)

S3 Fig D- and L-lactic acid content (% w/v) in CVM samples from (A) Group 1 (Nugent 0–3, high D); (B) Group 2 (Nugent 0–3, low D); (C) Group 3 (Nugent 4–6, intermediate) and (D) Group 4 (Nugent 7–10, BV). Data plotted as mean ± SEM. A value of zero indicates that the isomer lactic acid content was below the limit of detection for the assay.
(TIF)

S4 Fig CVM sample pH separated by Group and color-coded based on available 16S sequencing community state types. Data represented as mean ± SEM. **** p < 0.0001.
(TIF)

S5 Fig HIV virion ensemble-averaged mean square displacement <MSD> at a time scale (*τ*) of 1 s, where each data marker represents an individual CVM sample. Samples are grouped by participants that self-reported no birth control use (None), all participants that reported use of birth control (All BC), and then broken up into groups based on which birth control method was reported. Data represented as geometric mean and geometric mean standard deviation. Repeat samples from the same participant were excluded in statistical analyses.
(TIF)

S6 Fig Additional representative scanning electron microscopy (SEM) images of (A,B) Group 1; (C,D) Group 4 CVM samples. Scale bar = 1 μm. Each SEM image is from an individual participant.
(TIF)

S7 Fig HIV virion ensemble-averaged mean square displacement <MSD> at a time scale (*τ*) of 1 s in CVM samples obtained from participants before (pre) and after (post) antibiotic treatment (n = 4). Three women experienced resolution of their BV as defined by low Nugent (Nugent = 0) and pH < 4.5 (average 4.1 ± 0.6). The D-LA concentrations in these 3 CVM samples (average 0.03 ± 0.05% w/v) were consistent with *L*. *iners*-dominated (Group 2) microbiota. The CVM sample of the fourth participant had an intermediate Nugent score of 5 and pH of 5.22, suggesting either non-resolution or relapse to polymicrobial microbiota. The solid lines connect each participant’s pre and post samples, and the red dot indicates the post antibiotic treatment sample with a Nugent score of 5. The dashed line indicates the geometric mean <MSD> for Group 1 samples shown in Fig 2.
(TIF)

S1 Video Representative video of mCherry fluorescent HIV-1 virions “trapped” in a *L. crispatus*-dominant CVM sample. The time-lapsed video was obtained using an epifluorescence microscope at 15 Hz. The first 100 frames (~6–7 s) is displayed in real time.
(AVI)

S2 Video Representative video of mCherry fluorescent HIV-1 virions freely diffusing in a polymicrobial CVM sample. The time-lapsed video was obtained using an epifluorescence microscope at 15 Hz. The first 100 frames (~6–7 s) is displayed in real time.
(AVI)

S1 Table Participant CVM pH and percent water (% w/w) comparing the quick (<30 s) and 2 h Softcup insertion. (TIF)

S2 Table 2 X 2 contingency table for the assessment of sensitivity, specificity, positive predictive value and negative predictive value of Nugent score diagnosis of BV in relation to clustering based on 16S rRNA sequencing as the reference standard. (TIF)

S3 Table Particle size (Z ave.) and ζ-potential. Data represented as average ± SEM.
(TIF)

S1 Method Method for Softcup Timing Experiment. (DOCX)
