# Supplementary figures and images for: The cervicovaginal mucus barrier to HIV-1 is diminished in bacterial vaginosis
Source: PLoS Pathog. 2020 Jan 23;16(1):e1008236. doi: 10.1371/journal.ppat.1008236 (PMC6999914; doi:10.1371/journal.ppat.1008236)

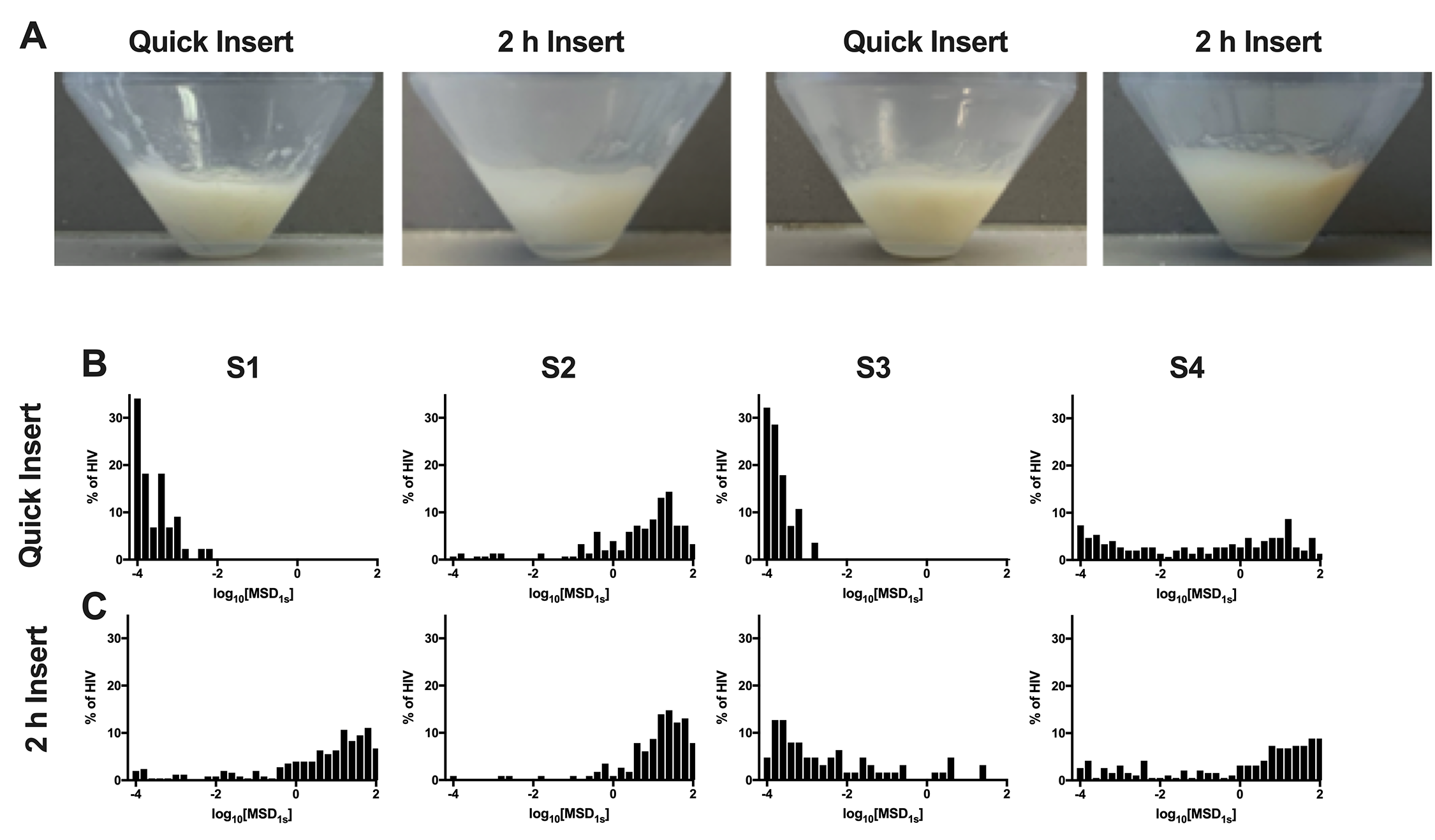

Supplement: S1 Fig — Individual virion data from each sample was binned and the percentage of total virions in each bin is displayed. Data for each participant is shown individually, as there was significant variability in the fraction of virions trapped in the samples with the Quick Insert. Overall, the 2 h insertion led to an increase in virion mobility in CVM for all participants (n = 4 participants, S1-4). (TIF) [file ppat.1008236.s001.tif]

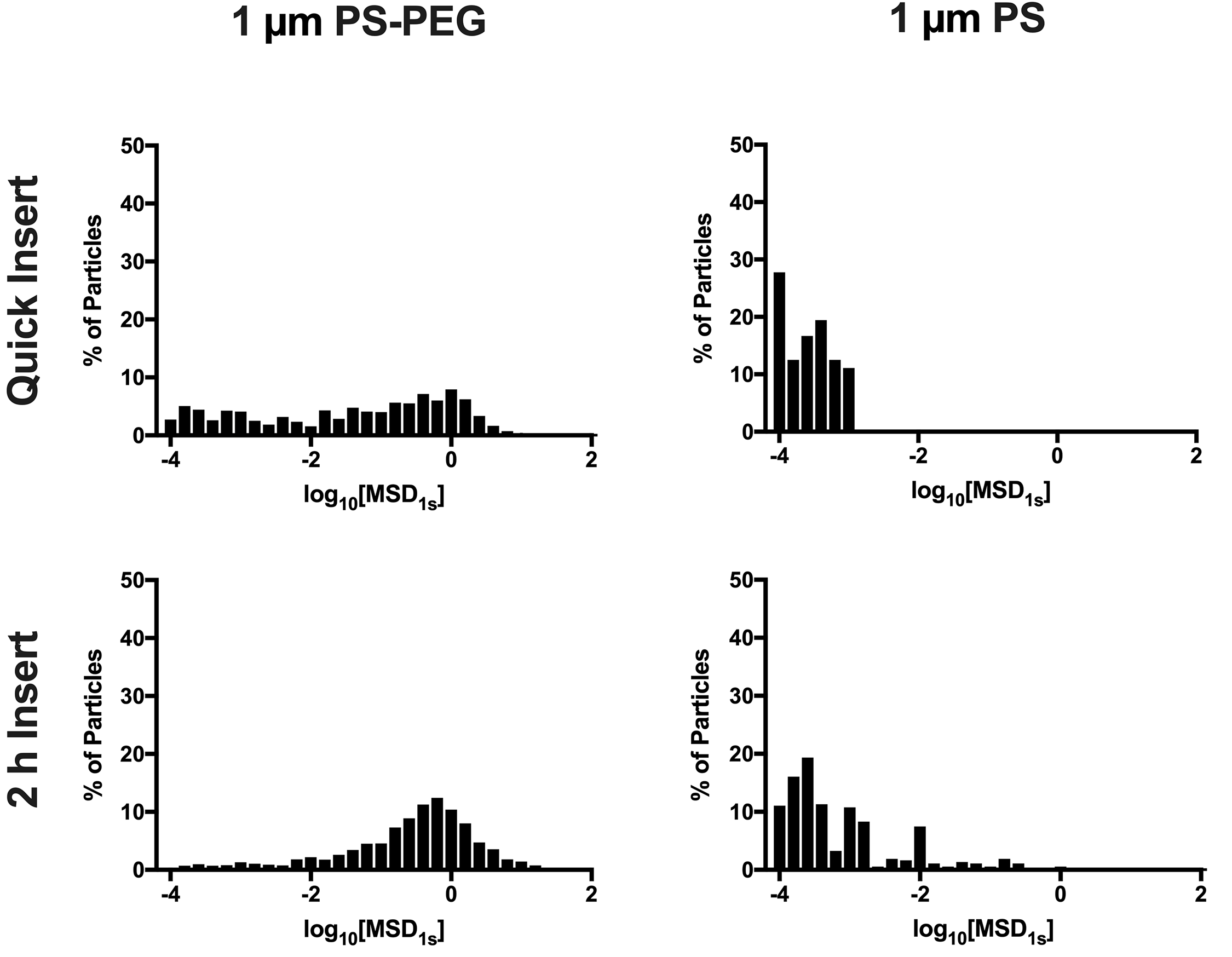

Supplement: S2 Fig — Individual particle data from each sample was binned, and the percentage of total particles in each bin was averaged over n = 3 participants. (TIF) [file ppat.1008236.s002.tif]

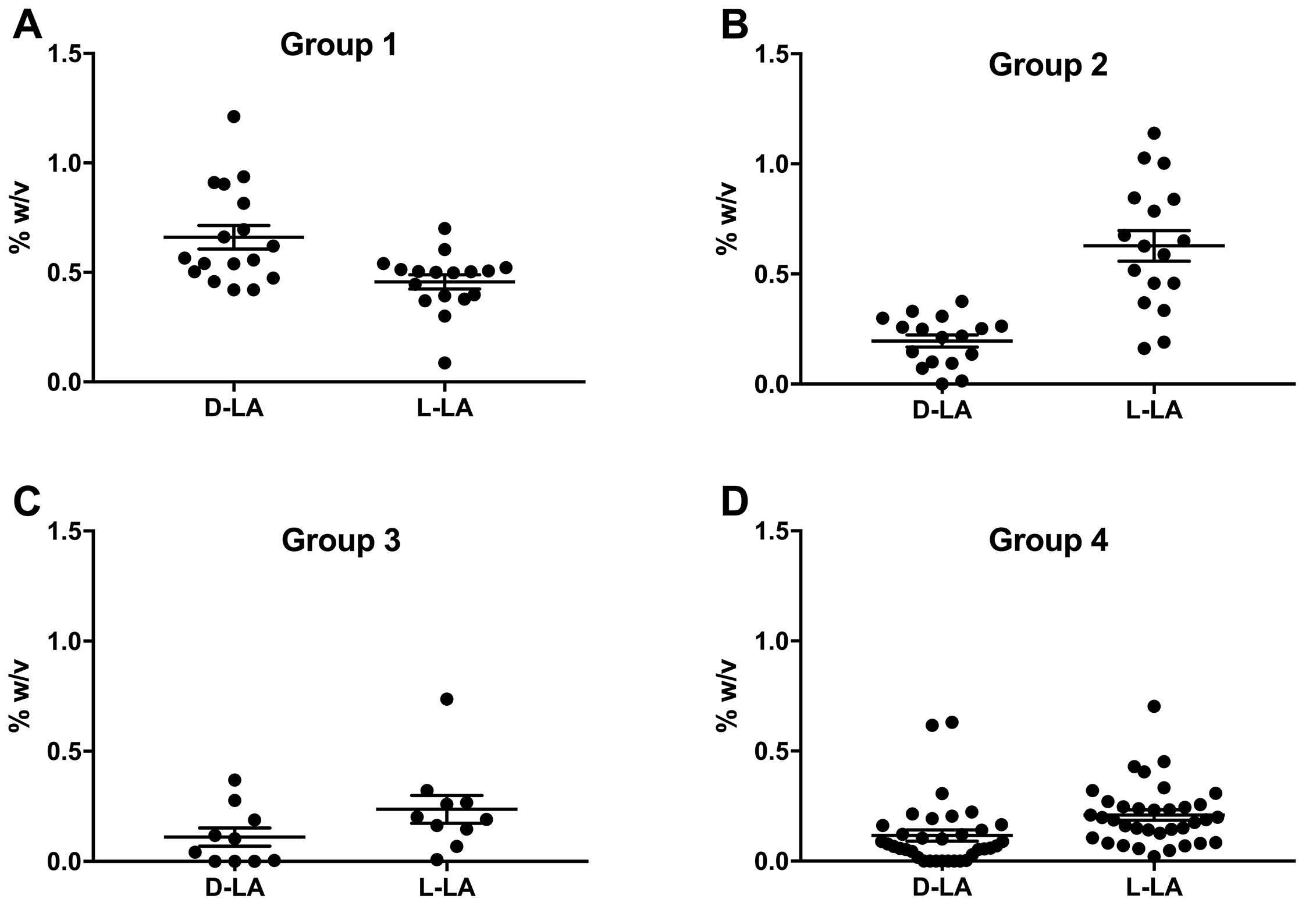

Supplement: S3 Fig — D- and L-lactic acid content (% w/v) in CVM samples from (A) Group 1 (Nugent 0–3, high D); (B) Group 2 (Nugent 0–3, low D); (C) Group 3 (Nugent 4–6, intermediate) and (D) Group 4 (Nugent 7–10, BV). Data plotted as mean ± SEM. A value of zero indicates that the isomer lactic acid content was below the limit of detection for the assay. (TIF) [file ppat.1008236.s003.tif]

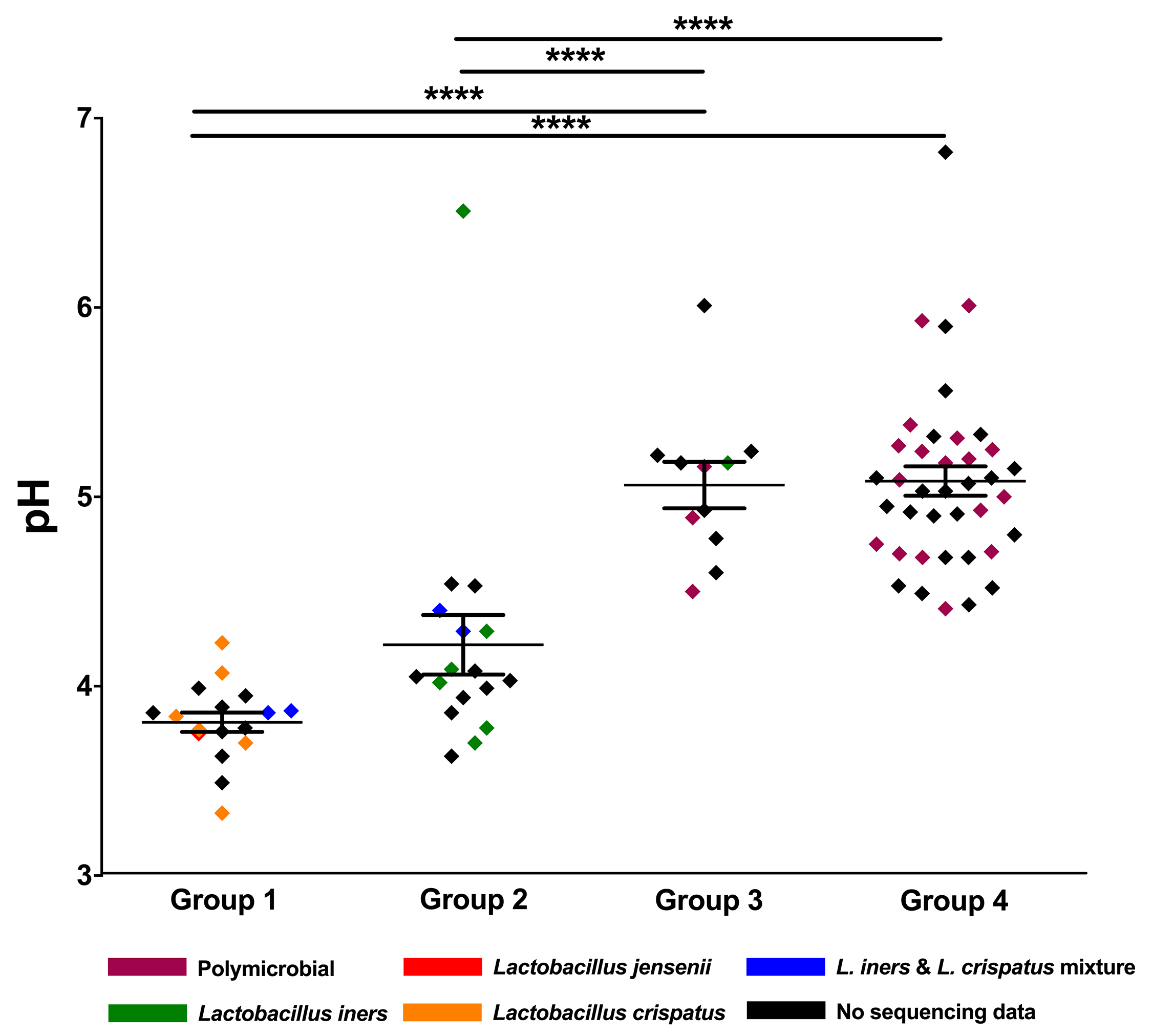

Supplement: S4 Fig — Data represented as mean ± SEM. **** p < 0.0001. (TIF) [file ppat.1008236.s004.tif]

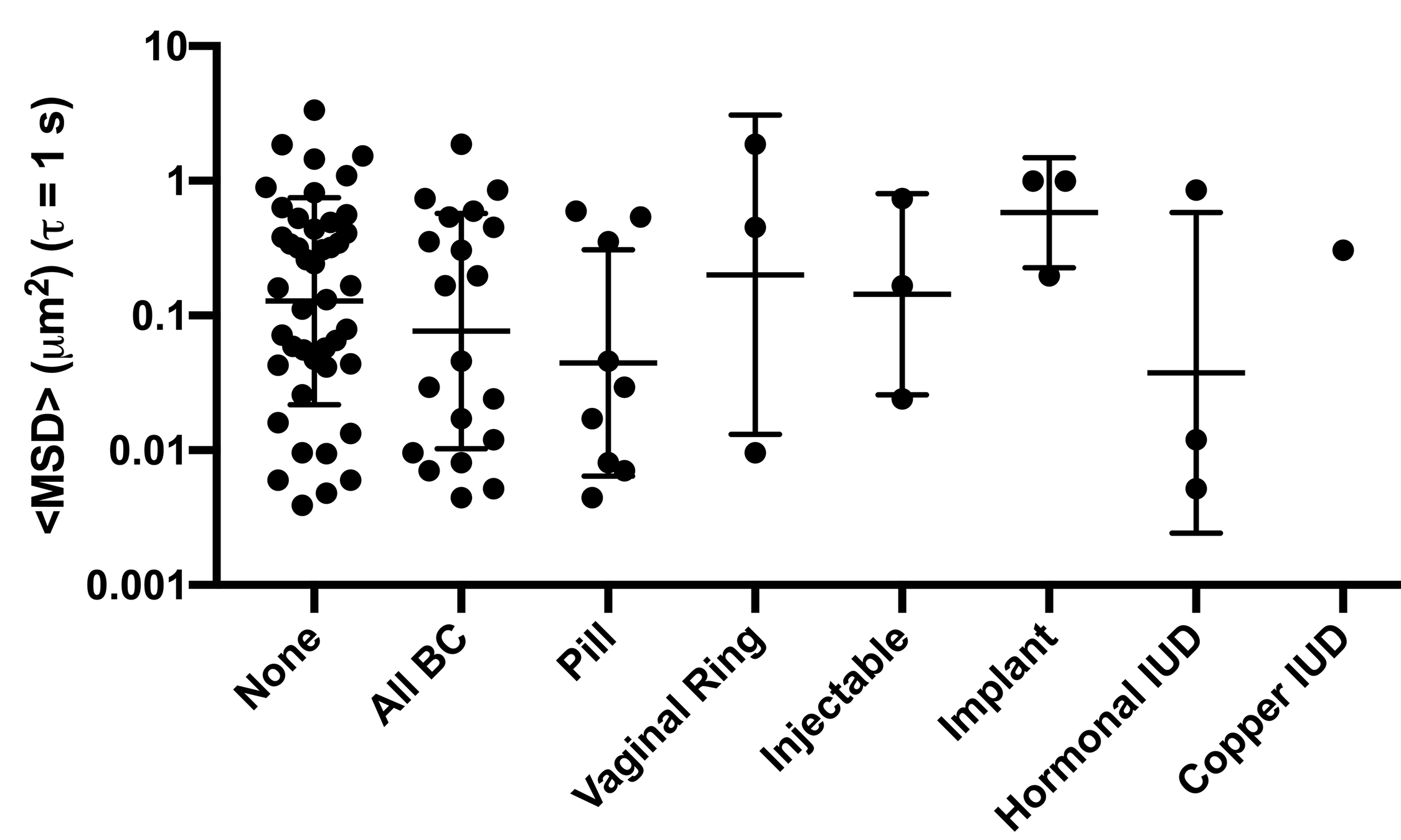

Supplement: S5 Fig — Samples are grouped by participants that self-reported no birth control use (None), all participants that reported use of birth control (All BC), and then broken up into groups based on which birth control method was reported. Data represented as geometric mean and geometric mean standard deviation. Repeat samples from the same participant were excluded in statistical analyses. (TIF) [file ppat.1008236.s005.tif]

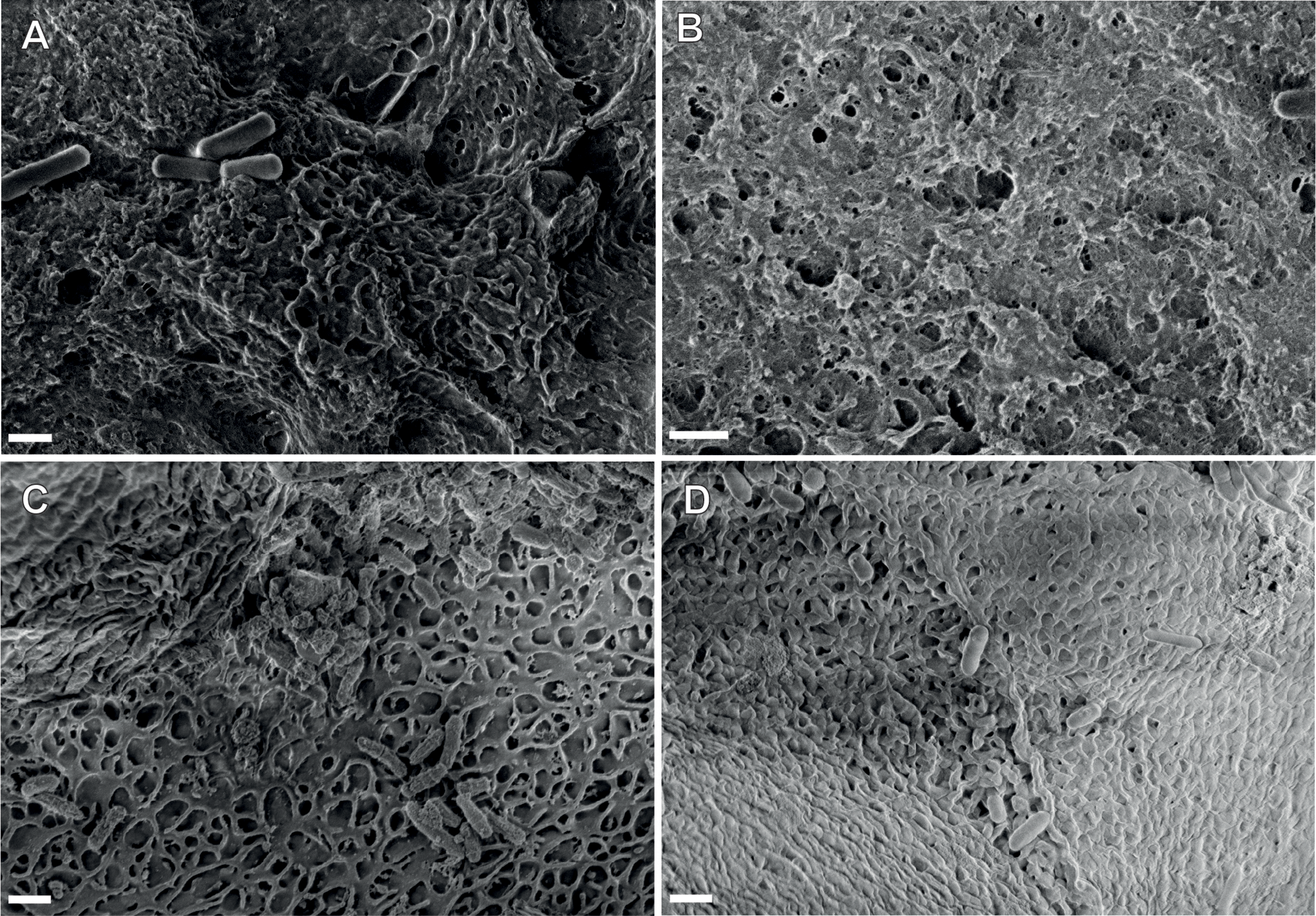

Supplement: S6 Fig — Additional representative scanning electron microscopy (SEM) images of (A,B) Group 1; (C,D) Group 4 CVM samples. Scale bar = 1 μm. Each SEM image is from an individual participant. (TIF) [file ppat.1008236.s006.tif]

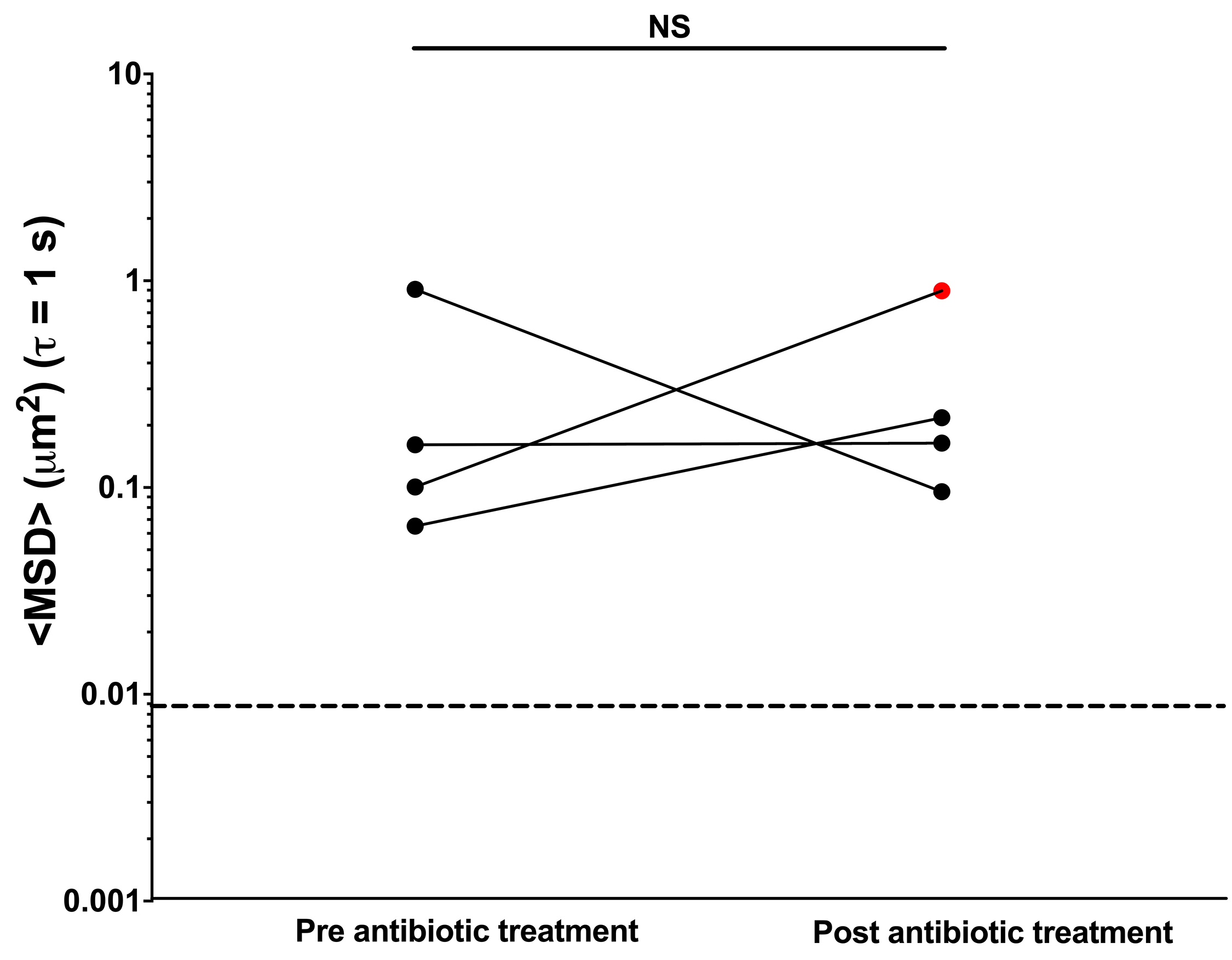

Supplement: S7 Fig — Three women experienced resolution of their BV as defined by low Nugent (Nugent = 0) and pH < 4.5 (average 4.1 ± 0.6). The D-LA concentrations in these 3 CVM samples (average 0.03 ± 0.05% w/v) were consistent with L. iners-dominated (Group 2) microbiota. The CVM sample of the fourth participant had an intermediate Nugent score of 5 and pH of 5.22, suggesting either non-resolution or relapse to polymicrobial microbiota. The solid lines connect each participant’s pre and post samples, and the red dot indicates the post antibiotic treatment sample with a Nugent score of 5. The dashed line indicates the geometric mean for Group 1 samples shown in Fig 2. (TIF) [file ppat.1008236.s007.tif]

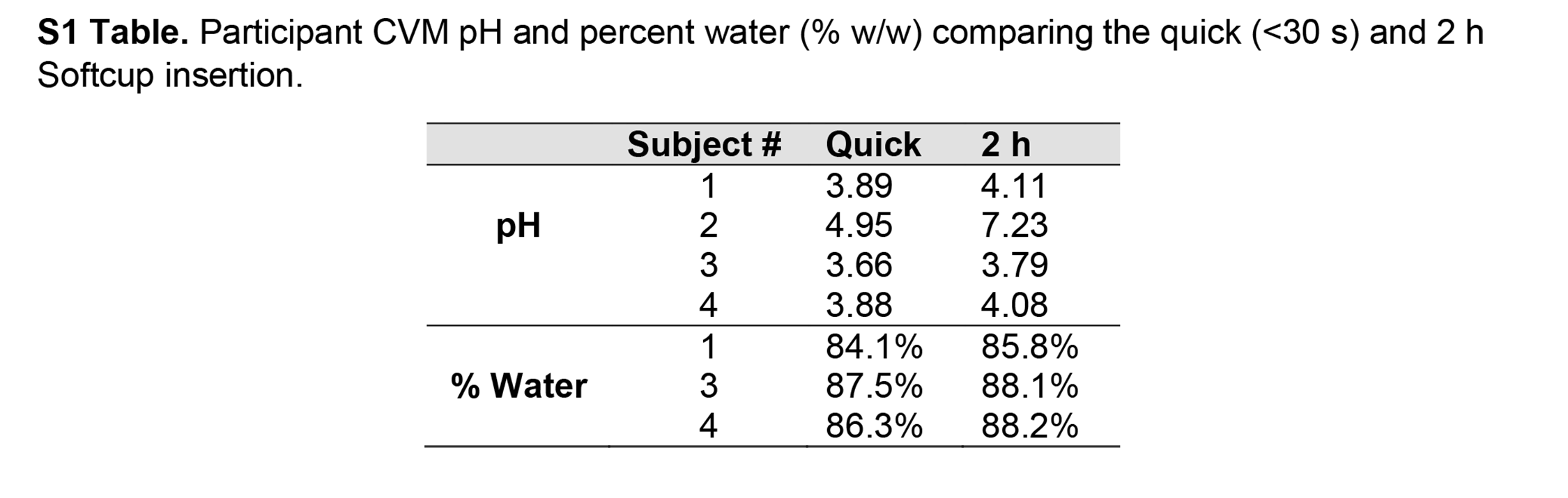

Supplement: S1 Table — (TIF) [file ppat.1008236.s010.tif]

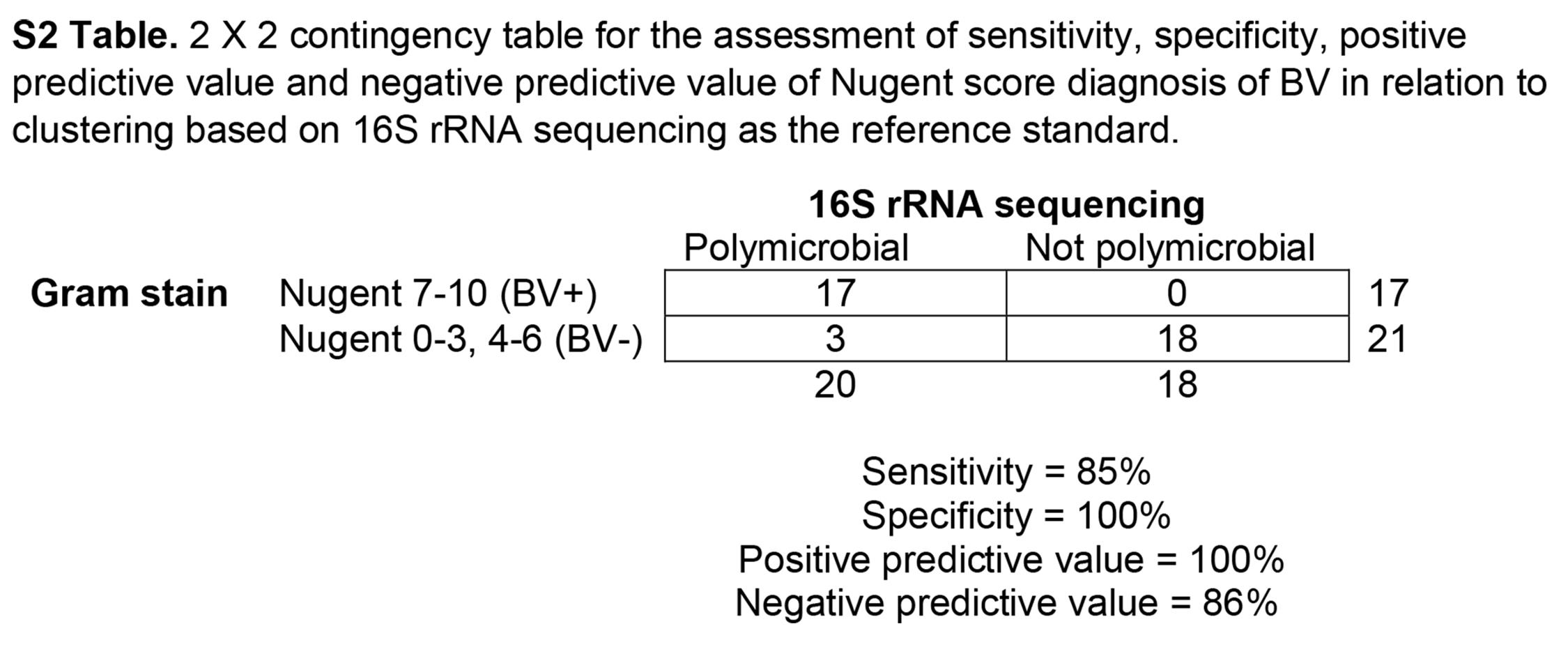

Supplement: S2 Table — (TIF) [file ppat.1008236.s011.tif]

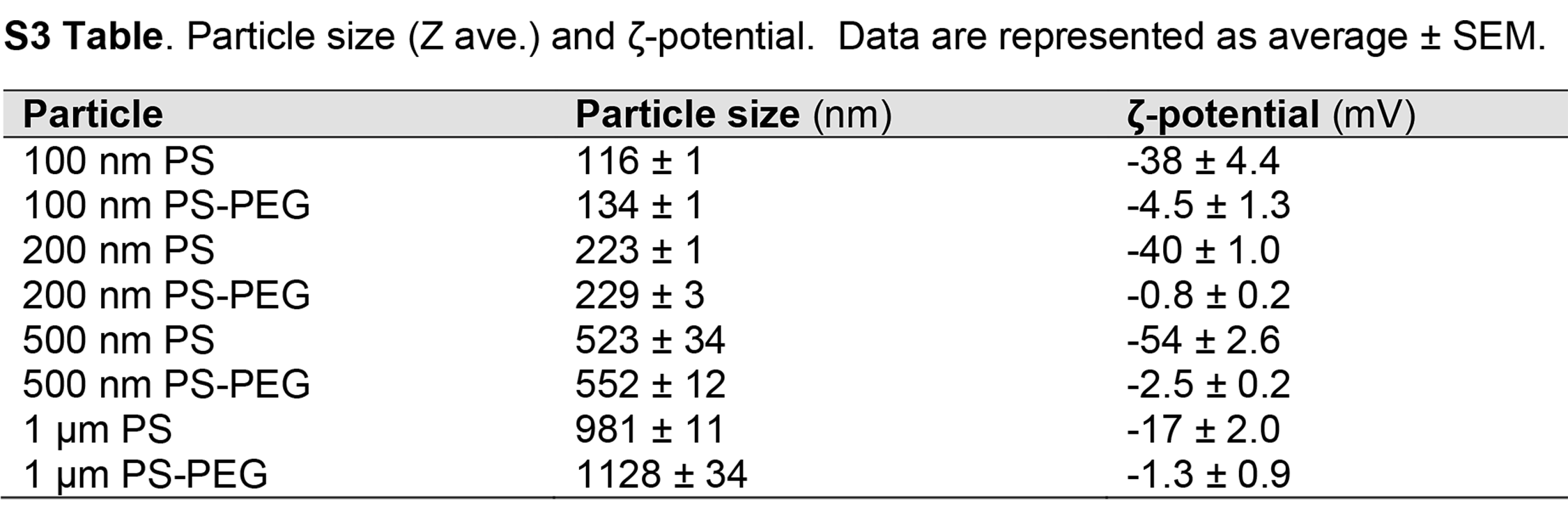

Supplement: S3 Table — Data represented as average ± SEM. (TIF) [file ppat.1008236.s012.tif]
