# Supplementary material for: The cervicovaginal mucus barrier to HIV-1 is diminished in bacterial vaginosis
Source: PLoS Pathog. 2020 Jan 23;16(1):e1008236. doi: 10.1371/journal.ppat.1008236 (PMC6999914; doi:10.1371/journal.ppat.1008236)
Supplement: S1 Method — (DOCX) [file ppat.1008236.s013.docx]

**Supporting Information Methods**

**Method for Softcup Timing Experiment**

To compare collection methods, participants were directed to collect two sequential CVM samples on the same day to avoid potential daily variation due to menstrual cycle and other factors. The first sample was denoted as the “Quick Insert”. Participants were instructed to insert the Softcup into their vagina and twist it 180˚ while removing it to collect undiluted CVM from the vaginal wall. The second sample was denoted as the “2 h Insert”. For this sample, participants were directed to insert the Softcup under the cervix as if they were using it for menstrual fluid collection and leave it in place for 2 h before removal. CVM samples were stored at 4°C and processed within 6 h of collection. Samples were characterized using Amsel’s criteria (ii)-(iv) and the pH was measured as described in the Materials and Methods in the main text. A portion of the sample was placed in a separate Eppendorf tube, weighed, and lyophilized for 48 h to determine the percentage of water content by weight. The percent of water content was calculated by subtracting the dry weight from the wet weight and dividing by the wet weight (([weight wet-dry weight]/wet weight)*100). Fluorescently labeled HIV virions,1 µm polystyrene (PS) particles, or 1 µm polyethylene glycol (PEG)-coated PS particles nanoparticles (0.4 µL) were added to 5 µL of undiluted CVM in custom-made glass slides, mixed, and immediately sealed with a glass coverslip. Multiple particle tracking was conducted as described in the Materials and Methods in the main text.
